# Fractional Factorial Design-Guided Optimization of Flavonoid Content and Antioxidant Capacity in *Justicia gendarussa* Leaf Extracts and UHPLC-HRMS-Based Metabolite Characterization

**DOI:** 10.3390/ph19091466

**Published:** 2026-09-16

**Authors:** Waras Nurcholis, Gendhis Mahestri Handiani, I Made Artika, Syarifah Iis Aisyah, Gyath Karadsheh, Aditya Kurniawan, Viktória Poór, Tamás Marosvölgyi, Ulfa Elfiah, Endre Kristóf, Rini Arianti

**Affiliations:** 1 Department of Biochemistry, Faculty of Mathematics and Natural Sciences, IPB University, Bogor 16680, Indonesia; gendhismahetri@apps.ipb.ac.id (G.M.H.); imart@apps.ipb.ac.id (I.M.A.); 2 Tropical Biopharmaca Research Center, IPB University, Bogor 16128, Indonesia; syarifah@apps.ipb.ac.id; 3 Department of Biochemistry and Molecular Biology, Faculty of Medicine, University of Debrecen, H-4032 Debrecen, Hungary; gyathkaradsheh@med.unideb.hu (G.K.); kurniawan.aditya@med.unideb.hu (A.K.); kristof.endre@med.unideb.hu (E.K.); ariantirini@med.unideb.hu (R.A.); 4 Doctoral School of Molecular Cellular and Immune Biology, University of Debrecen, H-4032 Debrecen, Hungary; 5 Doctoral School of Medical Sciences, University of Debrecen, H-4032 Debrecen, Hungary; 6 Department of Biology Education, Faculty of Education, University of Jember, Jember 68121, Indonesia; 7 Institute of Bioanalysis, Medical School, University of Pécs, H-7624 Pécs, Hungary; viktoria.poor@aok.pte.hu (V.P.); marosvolgyi.tamas@pte.hu (T.M.); 8 Szentágothai Research Center, University of Pécs, H-7624 Pécs, Hungary; 9 Plastic, Reconstructive and Aesthetic Surgery Division, Department of Surgery and Laboratory of Anatomy, Faculty of Medicine, University of Jember, Jember 68121, Indonesia; ulfa.fk@unej.ac.id

**Keywords:** *Justicia gendarussa*, extraction optimization, fractional factorial design, total flavonoid content, antioxidant capacity, DPPH, FRAP, CUPRAC, UHPLC-Q-Orbitrap HRMS, metabolite profiling

## Abstract

**Background/Objectives:** Extraction conditions can influence the chemical profile and antioxidant properties of medicinal plant extracts. This study aimed to optimize ethanol-based extraction of *Justicia gendarussa* leaves and characterize the optimized extract through UHPLC-HRMS metabolite profiling, with application to cultivated plant materials. **Methods:** A two-level half-fractional factorial design was used to assess the effects of five extraction factors—liquid-to-solid ratio, temperature, ethanol concentration, extraction time, and pH—on three response variables: total flavonoid content (TFC), DPPH radical-scavenging activity, and ferric reducing antioxidant power (FRAP). The optimized condition was validated experimentally, characterized by UHPLC-Q Exactive Plus Orbitrap HRMS with data-dependent MS/MS, and applied to cultivated leaf and stem materials under six fertilization treatments, analyzed using two-way ANOVA (organ × treatment). **Results:** The liquid-to-solid ratio × temperature interaction was the dominant determinant of TFC, and the liquid-to-solid ratio alone dominated both DPPH and FRAP. Desirability-based optimization identified a condition of 100 mL g^−1^ liquid-to-solid ratio, 30 °C, 20% ethanol, 30 min, and pH 2. Confirmatory extraction resulted in 16.43 ± 0.30 mg QE g^−1^ DW TFC, 12.30 ± 0.02 µmol TE g^−1^ DW DPPH, and 190.56 ± 2.58 µmol TE g^−1^ DW FRAP, in close agreement with model predictions (0.10–1.54% error). UHPLC-HRMS/MS detected 52 putatively annotated metabolites, including a candidate gendarusin A/B (apigenin di-*C*-arabinoside)-type *C*-glycosylflavone. Leaves showed significantly higher TFC, CUPRAC, and FRAP than stems regardless of fertilization treatment (organ effect *p* < 0.0001), whereas the leaf–stem difference in DPPH capacity varied significantly with treatment (interaction *p* = 0.0010). **Conclusions:** These findings provide a validated extraction condition for the measured responses and support further targeted metabolite evaluation of *J. gendarussa*.

## 1. Introduction

Flavonoid-rich plant extracts are widely studied for their antioxidant properties and functional value in food, pharmaceutical, and nutraceutical applications [1,2,3]. Extraction conditions, such as solvent composition, temperature, time, liquid-to-solid ratio, and pH, strongly influence solubility, stability, and ultimately the flavonoid content and antioxidant activity measured in an extract [4,5,6,7,8,9].

*Justicia gendarussa* Burm. f., a Southeast Asian medicinal plant, contains alkaloids, flavonoids, phenolics, saponins, aromatic amines, and fatty acids with antioxidant, antihyperglycemic, antidiarrheal, and antimicrobial relevance [10,11]. Prior work showed that solvent choice affects phenolic and flavonoid content, antioxidant activity, and UPLC-MS/MS metabolite profiles [12], and that ethanol concentration, temperature, and time jointly influence phenolic recovery [13]. These studies leave open how several extraction variables behave together when flavonoid recovery and multiple antioxidant responses are evaluated within one experimental design.

Extraction variables rarely act independently: the effect of ethanol concentration can depend on pH, since phenolic polarity and stability vary with the extraction medium [14], and the effect of temperature can depend on time or solvent volume through their joint influence on diffusion and degradation [15]. A one-factor-at-a-time approach is therefore limited for this type of investigation [16]. Fractional factorial design (FFD) allows several variables to be examined simultaneously with a manageable number of runs [17]. In this study, five variables—liquid-to-solid ratio, temperature, ethanol concentration, extraction time, and pH—were evaluated in a two-level half-FFD; pH was included given its documented relevance to alkaloid removal and flavonoid stability in *J. gendarussa* preparations [10].

Measured antioxidant activity also depends on the assay used. Total flavonoid content (TFC) captures a broad compound class but does not necessarily predict activity across different mechanisms. 2,2-diphenyl-1-picrylhydrazyl (DPPH) and ferric reducing antioxidant power (FRAP) were used during extraction optimization, whereas cupric reducing antioxidant capacity (CUPRAC) was included in the subsequent evaluation of cultivated leaf and stem samples; these assays respectively estimate radical-scavenging capacity, ferric-reducing power, and cupric-reducing capacity through the Cu(II)-neocuproine system [18,19,20], and extracts can be ranked differently across them because each responds to different compound structures and redox mechanisms [21,22,23]. A condition selected from a single response may therefore not balance flavonoid recovery with antioxidant capacity.

Chemical profiling is also needed to characterize what is recovered under the selected condition, since TFC alone cannot account for the contribution of non-flavonoid constituents to antioxidant activity. Ultra-high-performance liquid chromatography coupled to quadrupole-Orbitrap high-resolution mass spectrometry (UHPLC-Q-Orbitrap HRMS) can provide a broader view of the metabolite classes present, although annotations lacking authentic standards should be treated as putative [24,25]. An optimized method’s practical value also depends on its applicability to cultivated material: fertilization regime and plant organ can influence flavonoid accumulation and antioxidant capacity, and nitrogen-related cultivation has been reported to affect *J. gendarussa* metabolite profiles and antioxidant capacity [26,27,28].

Therefore, this study optimized ethanol-based extraction of *J. gendarussa* leaves using a two-level FFD, with TFC, DPPH radical-scavenging capacity, and FRAP as outputs. The selected extraction condition was validated experimentally and applied to UHPLC-Q-Orbitrap HRMS-based metabolite profiling. Finally, leaf and stem samples from cultivated *J. gendarussa* grown under different fertilization regimes were analyzed—linking extraction optimization, chemical profiling, and cultivated-sample evaluation as a basis for improving the quality assessment of *J. gendarussa* materials.

## 2. Results

### 2.1. Experimental Matrix and Observed Responses

The extraction efficiency of bioactive secondary metabolites from *J. gendarussa* was systematically investigated using a FFD, evaluating five critical process parameters: liquid-to-solid ratio (A), temperature (B), ethanol concentration (C), extraction time (D), and pH (E). As summarized in the experimental matrix (Table 1), the 19 independent runs revealed profound variability across all responses—TFC, DPPH radical-scavenging capacity, and FRAP. These fluctuations indicate that the phytochemical recovery of *J. gendarussa* is highly sensitive to extraction conditions, particularly to the combined effects of solvent polarity, thermal input, and extraction-medium acidity.

The recovery of TFC exhibited a wide distribution, ranging from 3.02 ± 0.247 to 25.57 ± 0.165 mg quercetin equivalent (QE) g^−1^ dry weight (DW). The minimum TFC yield was recorded in Run 18, performed at 100 mL g^−1^, 80 °C, 20% ethanol, 300 min, and pH 2, whereas the maximum was achieved in Run 17, performed at 100 mL g^−1^, 30 °C, 20% ethanol, 300 min, and pH 6. Although both runs used a high liquid-to-solid ratio and prolonged extraction time, their markedly different TFC values suggest that elevated temperature under strongly acidic conditions may compromise the stability or extractability of specific flavonoid constituents in *J. gendarussa*.

Antioxidant capacities also varied markedly among the experimental runs. DPPH radical-scavenging activity ranged from 0.13 ± 0.004 to 26.64 ± 0.270 µmol Trolox equivalent (TE) g^−1^ DW, with the highest value observed in Run 5. FRAP values showed an even broader range, from 19.45 ± 0.160 to 242.41 ± 0.769 µmol TE g^−1^ DW. Interestingly, Run 18 produced the highest FRAP value despite yielding the lowest TFC. This divergence suggests that the compounds contributing to ferric reducing capacity are not necessarily the same compounds quantified by the aluminium chloride flavonoid assay. The comprehensive raw data presented in Table 1 provide the empirical basis for statistical modeling of the interacting extraction variables.

### 2.2. Statistical Modeling and Component Interactions

ANOVA was used to identify the dominant model terms and their relative contributions to response variance (Table 2). The interaction between liquid-to-solid ratio and temperature (A × B) emerged as the dominant determinant of TFC (*p* = 0.0005, 28.03% contribution), acting negatively—the positive effect of increasing ratio was attenuated at higher temperature. The main effect of liquid-to-solid ratio itself was also significant (A, *p* = 0.0010, 22.66% contribution), followed by the temperature × ethanol concentration interaction (B × C, *p* = 0.0044, 13.80% contribution).

DPPH radical-scavenging capacity was overwhelmingly dominated by a single factor rather than multifactorial interactions: the liquid-to-solid ratio (A, *p* < 0.0001, 79.65% contribution), with smaller contributions from the ratio × time interaction (A × D, *p* = 0.0125, 5.83% contribution) and time itself (D, *p* = 0.0298, 4.16% contribution). FRAP was likewise dominated by the liquid-to-solid ratio (A, *p* < 0.0001, 39.82% contribution), with meaningful contributions from extraction time (D, *p* = 0.0061, 9.22% contribution) and the ratio × ethanol concentration interaction (A × C, *p* = 0.0085, 8.07% contribution). Across all three responses, the liquid-to-solid ratio was therefore the single most influential extraction factor.

**Figure 1:**
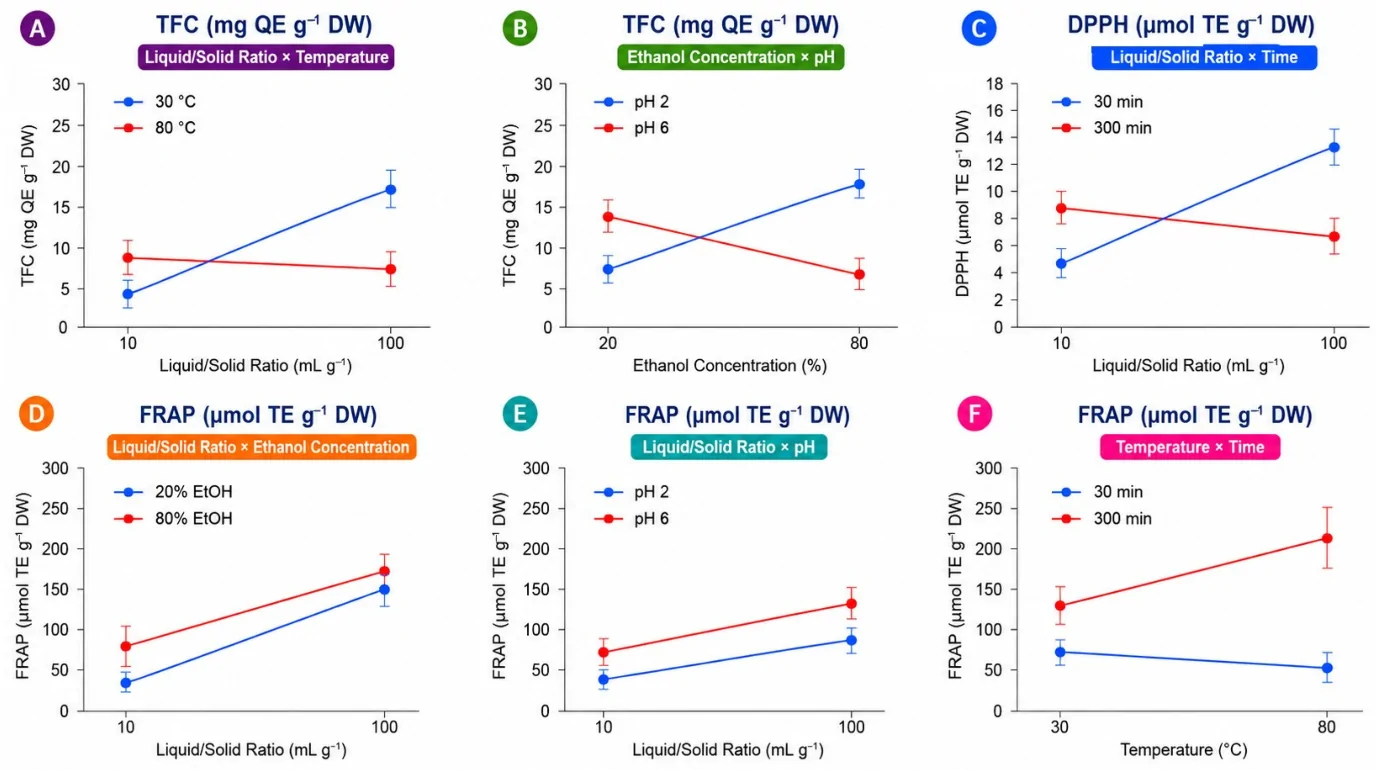
Significant two-factor interaction plots showing the effects of selected extraction variables on total flavonoid content (TFC) and antioxidant capacity in *Justicia gendarussa* leaf extracts. (**A**) Liquid-to-solid ratio × temperature for TFC. (**B**) Ethanol concentration × pH for TFC. (**C**) Liquid-to-solid ratio × time for 2,2-diphenyl-1-picrylhydrazyl (DPPH) radical-scavenging capacity. (**D**) Liquid-to-solid ratio × ethanol concentration for ferric reducing antioxidant power (FRAP). (**E**) Liquid-to-solid ratio × pH for FRAP. (**F**) Temperature × time for FRAP. Values represent model-predicted means generated from the corrected fractional factorial model.

The significant two-factor interaction plots in Figure 1 further illustrate these non-linear extraction patterns. For TFC, the A × B interaction shows that the positive effect of increasing liquid-to-solid ratio is the strongest at lower temperature, indicating that excessive solvent dilution combined with high temperature does not further improve flavonoid recovery. For DPPH, the A × D interaction indicates that the strong positive effect of liquid-to-solid ratio weakens somewhat at longer extraction times. These patterns confirm that a single-factor extraction approach would be insufficient to capture the complexity of *J. gendarussa* phytochemical recovery.

### 2.3. Multi-Response Optimization and Validation

To identify a standardized extraction protocol capable of simultaneously maximizing TFC, DPPH, and FRAP, a desirability-based multi-response optimization was performed. The top-ranked solutions are presented in Table 3. The best solution achieved an overall desirability of 0.719 and was defined by a liquid-to-solid ratio of 100 mL g^−1^, extraction temperature of 30 °C, ethanol concentration of 20%, extraction time of 30 min, and pH 2. Under these conditions, the model predicted a TFC of 16.451 mg QE g^−1^ DW, DPPH capacity of 12.112 µmol TE g^−1^ DW, and FRAP value of 191.279 µmol TE g^−1^ DW.

Experimental validation confirmed the reliability of the optimized model (Table 4). The observed values (mean ± SD of three technical analytical readings from a single confirmatory extraction) were 16.434 ± 0.303 mg QE g^−1^ DW for TFC, 12.299 ± 0.016 µmol TE g^−1^ DW for DPPH, and 190.558 ± 2.577 µmol TE g^−1^ DW for FRAP, corresponding to prediction errors of 0.10%, 1.54%, and 0.38%, respectively. All experimental values fell well within the 95% prediction intervals, indicating that the corrected FFD model provided an excellent prediction of extraction performance.

**Figure 2:**
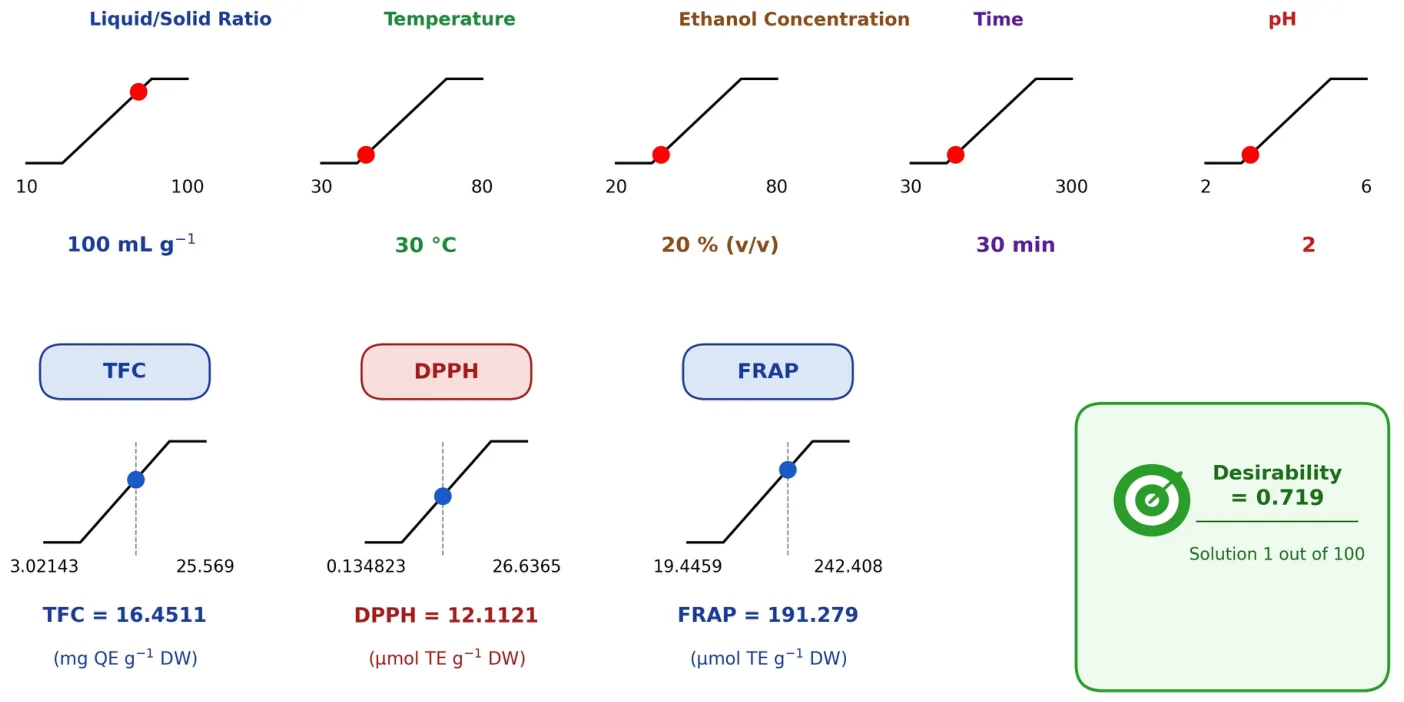
Desirability ramps for the numerical multi-response optimization displaying the selected level of each factor: liquid-to-solid ratio, temperature, ethanol concentration, time, pH (**top** panels) and the corresponding predicted value of each response: TFC, DPPH, FRAP (**bottom** panels) relative to its observed range, together with the overall desirability. The selected solution (desirability = 0.719) was achieved at a lower temperature and higher liquid-to-solid ratio, corresponding to predicted values of TFC (16.451 mg QE g^−1^ DW), DPPH (12.112 µmol TE g^−1^ DW), and FRAP (191.279 µmol TE g^−1^ DW).

The desirability ramps for each factor and response, which display how the selected solution satisfies the optimization criteria simultaneously, were shown in Figure 2. The highest desirability was achieved under low-temperature, high-solvent-volume conditions, indicating that a larger liquid-to-solid ratio combined with lower temperature improved extraction efficiency, consistent with the negative A × B interaction identified for TFC. This validated condition was therefore selected for subsequent metabolite profiling and application to cultivated *J. gendarussa* samples.

### 2.4. UHPLC-Q-Orbitrap HRMS-Based Metabolite Profile of the Optimized Extract

**Figure 3:**
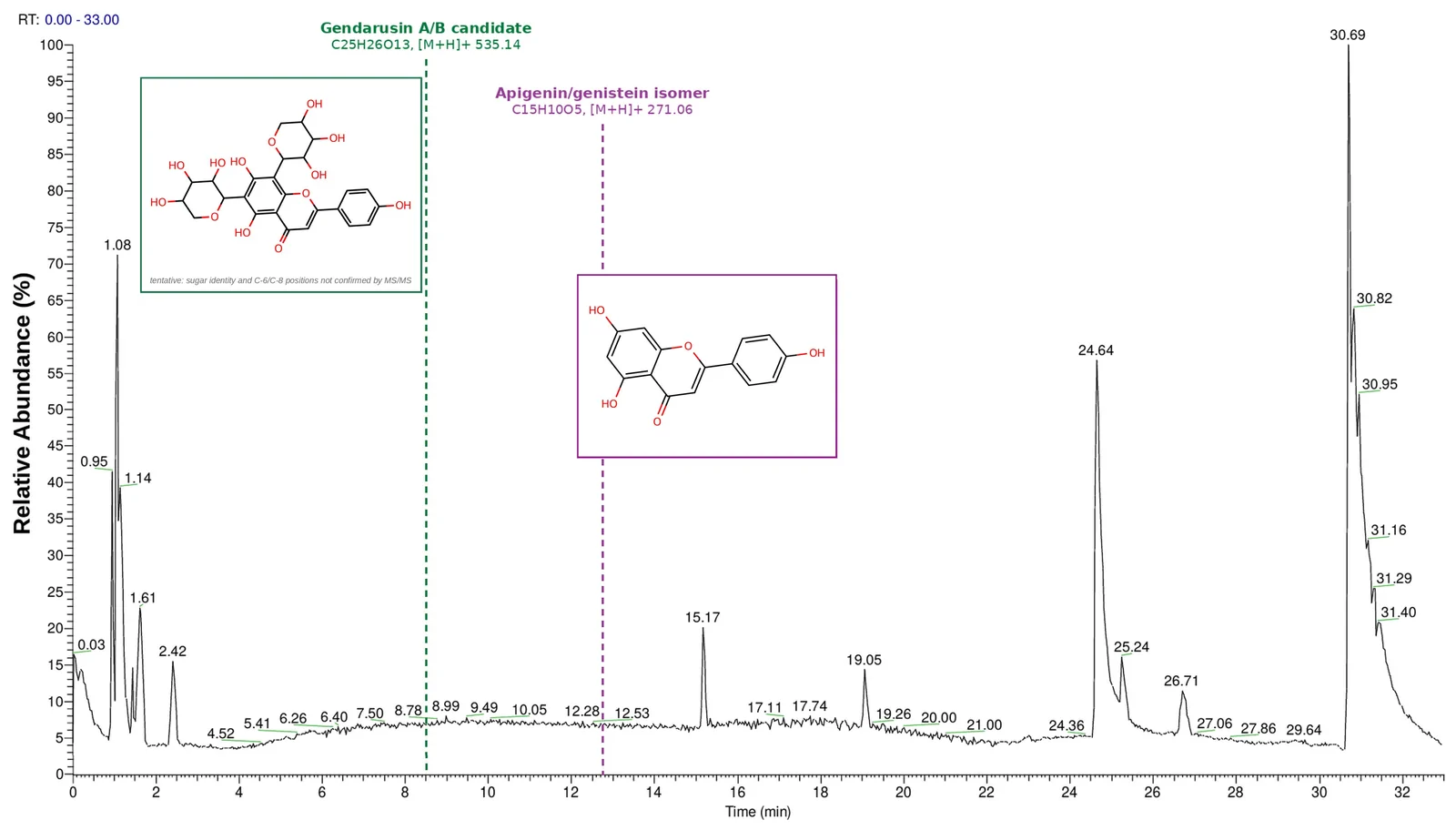
UHPLC-Q Exactive Plus Orbitrap high-resolution mass spectrometry base peak chromatogram (positive ionization mode) of the optimized *J. gendarussa* extract, prepared under the corrected optimal extraction condition (100 mL g^−1^ liquid-to-solid ratio, 30 °C, 20% ethanol, 30 min, pH 2). Dashed lines mark the retention times (RTs) of the two most chemotaxonomically relevant tentatively annotated metabolites: a candidate gendarusin A/B (apigenin di-*C*-arabinoside-type *C*-glycosylflavone, C_25_H_26_O_13_, [M + H]^+^ 535.14) at RT 8.52 min, and an apigenin/genistein-isomeric flavonoid (C_15_H_10_O_5_, [M + H]^+^ 271.06) at RT 12.77 min. Chemical structures are shown in colored inset boxes matching each dashed line (green: candidate apigenin di-*C*-pentosylflavone, with the *C*-glycoside regiochemistry and sugar identity shown as tentative since they are not confirmed by MS/MS alone; magenta: apigenin, the favored assignment for the second feature on chemotaxonomic grounds). The complete list of 52 putatively annotated metabolites, with RTs, is provided in Table 5.

The optimized extract was further characterized using UHPLC-Q-Orbitrap HRMS to determine the putative metabolite profile associated with the validated extraction condition. The chromatographic profile (positive ionization mode, data-dependent MS/MS) revealed 52 curated peaks distributed across a retention-time range of 0.95–28.36 min (Figure 3, Table 5). The early chromatographic region was dominated by highly polar compounds, including amino acids, sugar-related metabolites, and organic acids. Later-eluting peaks corresponded mainly to phenolic-related compounds, including a candidate gendarusin A/B (apigenin di-*C*-arabinoside)-type *C*-glycosylflavone, together with hydroxy fatty acids and oxygenated fatty acids or oxylipins.

Among the putatively annotated metabolites, L-valine showed the highest relative signal area (28.09%) within the curated feature set, followed by L-leucine (12.17%), DL-phenylalanine (6.98%), choline (5.69%), and diethylene glycol (4.89%). A candidate gendarusin A/B (apigenin di-*C*-arabinoside)-type *C*-glycosylflavone, tentatively identified from a previously unannotated feature (C_25_H_26_O_13_, RT 8.52 min), accounted for 2.52% of the relative signal area and is supported by diagnostic MS/MS fragmentation (sequential water losses and a cross-ring glycosidic fragment), though its specific regiochemistry could not be confirmed by MS/MS alone. An additional flavonoid feature (C_15_H_10_O_5_, RT 12.77 min, 0.31% relative signal area) is consistent with either apigenin or genistein; apigenin is favored on chemotaxonomic grounds, as genistein is a soy-associated isoflavone atypical of Acanthaceae [29]. The presence of amino acids, organic acids, and a diverse flavonoid/fatty-acid profile indicates that the corrected optimal extraction condition recovered a chemically diverse metabolite pool. All metabolites are reported as putatively annotated compounds and require confirmation using authentic standards for definitive identification.

### 2.5. Application of the Optimized Extraction Protocol to Cultivated J. gendarussa Samples

The validated extraction condition was subsequently applied to leaf and stem materials of cultivated *J. gendarussa* subjected to six fertilization regimes: T1, control without fertilizer; T2, 100% manure; T3, 100% Nitrogen, phosphorus, and potassium fertilizer (NPK); T4, 50% manure + 50% NPK; T5, 10% manure + 50% NPK; and T6, 50% manure + 10% NPK. This follow-up analysis was performed to evaluate whether the optimized extraction protocol could consistently recover flavonoids and antioxidant activity from plant materials produced under different cultivation conditions.

**Figure 4:**
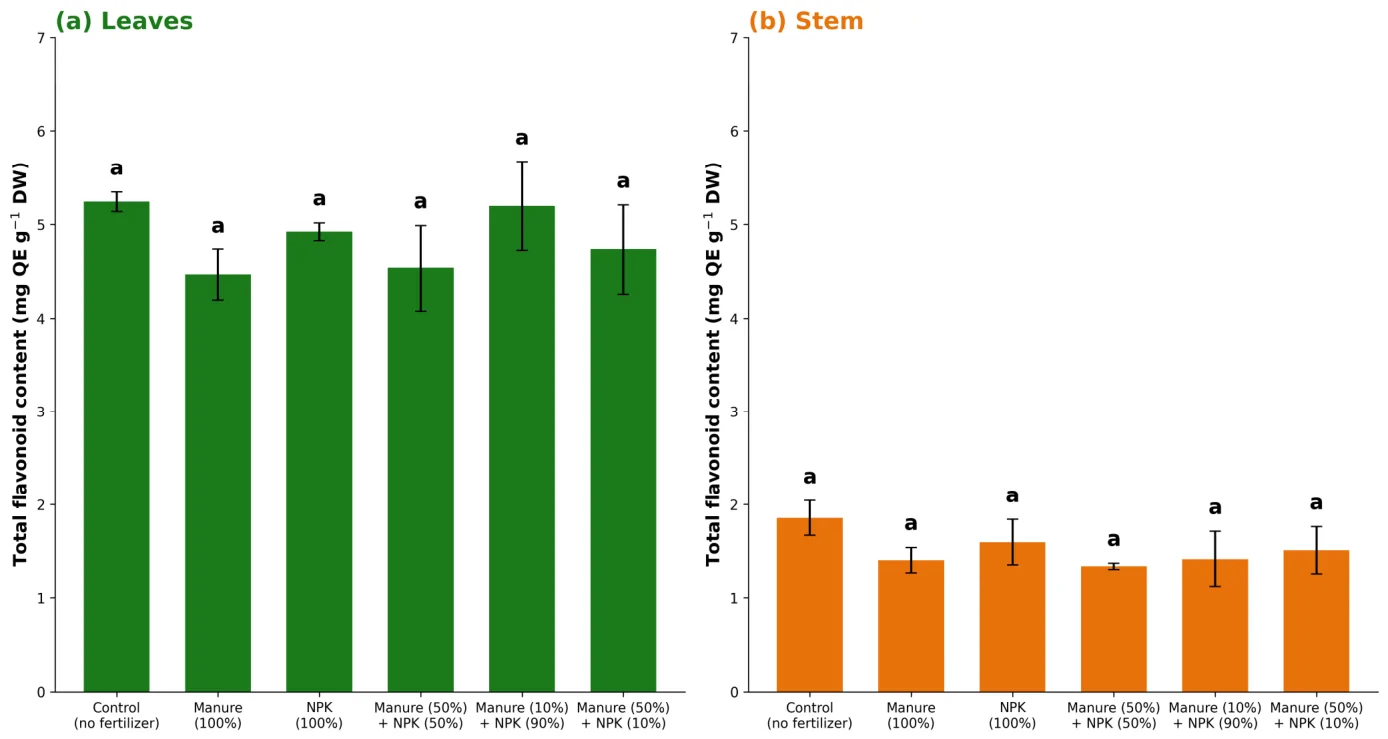
Total flavonoid content (TFC) of cultivated *J. gendarussa* leaf and stem extracts obtained using the corrected optimized extraction condition. T1, control without fertilizer; T2, 100% manure; T3, 100% NPK; T4, 50% manure + 50% NPK; T5, 10% manure + 50% NPK; T6, 50% manure + 10% NPK. Values are mean ± SEM (*n* = 3 independent extractions). Bars sharing the same letter within a panel are not significantly different (Tukey’s HSD, α = 0.05, pooled error term from a two-way organ × treatment ANOVA); leaves showed significantly higher TFC than stems overall (organ effect, *p* < 0.0001), consistently across all treatments (organ × treatment interaction not significant, *p* = 0.865).

TFC was consistently higher in leaves than in stems across all fertilization treatments (Figure 4). In leaves, TFC ranged from 4.46 ± 0.27 to 5.24 ± 0.10 mg QE g^−1^ DW, with the highest value observed in the unfertilized control treatment (T1). The T5 treatment also produced a comparable leaf TFC value of 5.19 ± 0.48 mg QE g^−1^ DW. In stems, TFC ranged from 1.34 ± 0.03 to 1.86 ± 0.19 mg QE g^−1^ DW, with the highest value again observed in T1. These results indicate that leaf tissue represents the major flavonoid-accumulating organ in cultivated *J. gendarussa*, whereas fertilization treatment did not result in significant differences in TFC within each organ. A two-way ANOVA (organ × fertilization treatment, using the per-replicate biological data) confirmed that organ was a highly significant determinant of TFC (*p* < 0.0001), while the treatment effect (*p* = 0.287) and the organ × treatment interaction (*p* = 0.865) were not significant, indicating that the leaf-over-stem TFC advantage was consistent across all six fertilization treatments.

**Figure 5:**
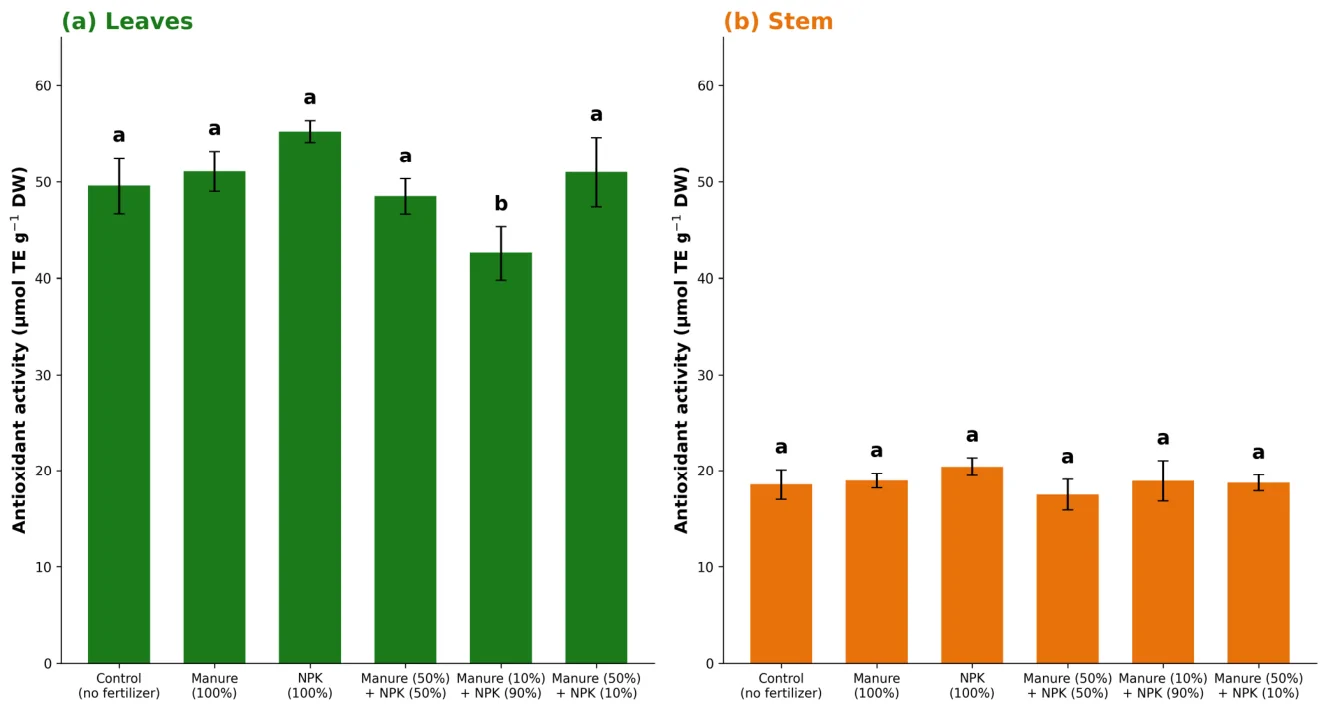
Cupric reducing antioxidant capacity (CUPRAC) antioxidant activity of cultivated *J. gendarussa* leaf and stem extracts obtained using the corrected optimized extraction condition. Treatment codes are as described in Figure 4. Values are mean ± SEM (*n* = 3 independent extractions). Bars sharing the same letter within a panel are not significantly different (Tukey’s HSD, α = 0.05, pooled two-way ANOVA error term). Within leaves, T5 was significantly lower than the other treatments; no significant treatment differences were detected within stems. Leaves showed significantly higher CUPRAC than stems overall (organ effect, *p* < 0.0001; organ × treatment interaction not significant, *p* = 0.166).

CUPRAC antioxidant capacity also showed organ-dependent differences (Figure 5). Leaf extracts had substantially higher CUPRAC values than stem extracts, ranging from 42.59 ± 2.80 to 55.25 ± 1.11 µmol TE g^−1^ DW. The highest leaf CUPRAC activity was observed in the 100% NPK treatment (T3), whereas the lowest was observed in T5. Stem CUPRAC activity was lower and ranged from 17.48 ± 1.58 to 20.40 ± 0.92 µmol TE g^−1^ DW, with the highest numerical value also detected in T3. These findings suggest that NPK fertilization may enhance reducing antioxidant capacity, particularly in leaf tissue. A two-way ANOVA confirmed organ as a highly significant determinant of CUPRAC (*p* < 0.0001); the treatment effect was marginally significant (*p* = 0.047), while the organ × treatment interaction was not significant (*p* = 0.166), indicating a consistent leaf-over-stem advantage across treatments. Within leaves specifically, Tukey’s HSD test identified T5 as having significantly lower antioxidant capacity than the other treatments; however, no significant treatment differences were detected within stems.

**Figure 6:**
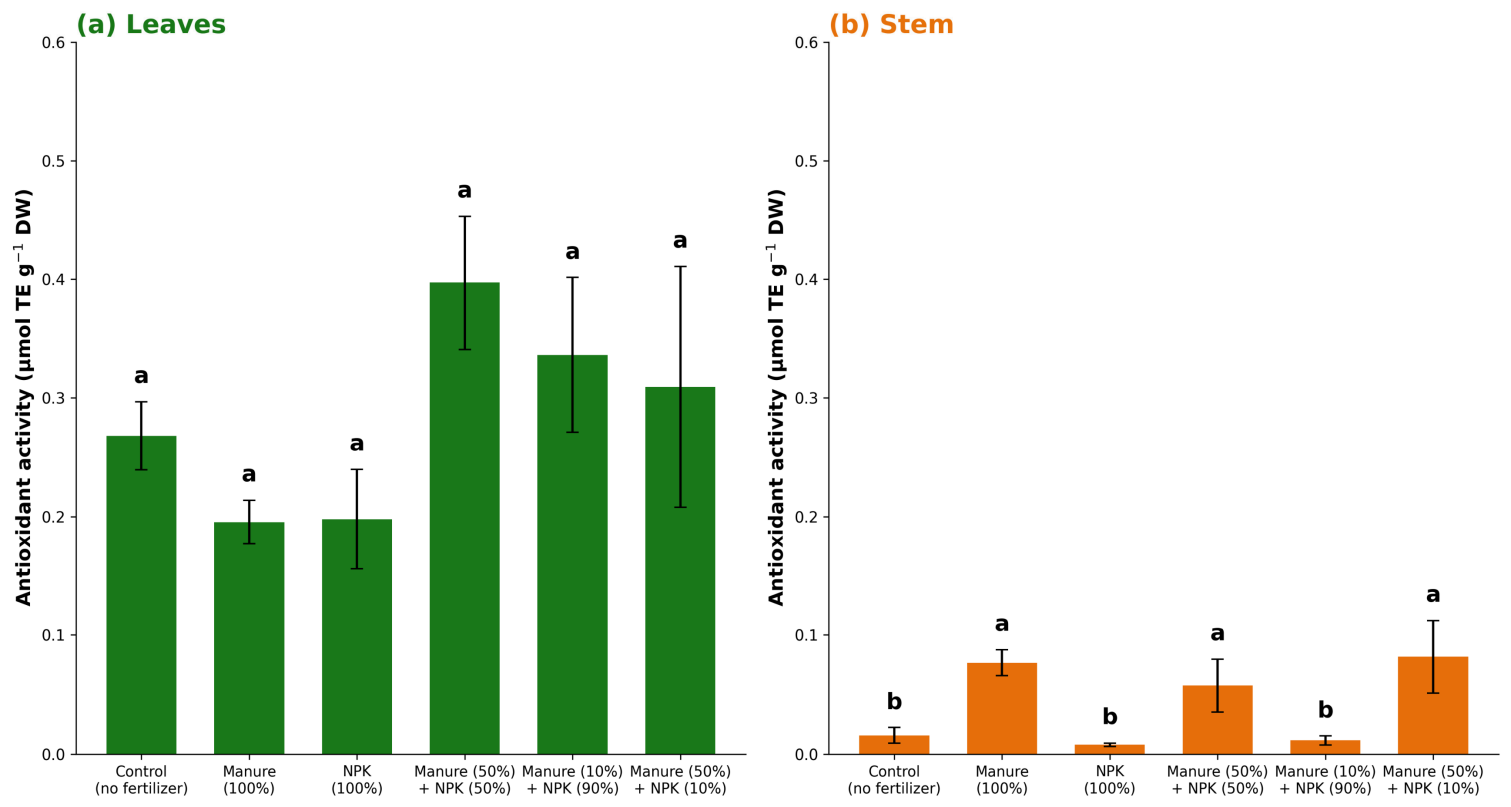
2,2-diphenyl-1-picrylhydrazyl (DPPH) radical-scavenging activity of cultivated *J. gendarussa* leaf and stem extracts obtained using the corrected optimized extraction condition. Treatment codes are as described in Figure 4. Values are mean ± SEM (*n* = 3 independent extractions); data were natural-log-transformed for statistical testing to meet the normality assumption. Bars sharing the same letter within a panel are not significantly different (Tukey’s HSD, α = 0.05, pooled two-way ANOVA error term). No significant treatment differences were detected within leaves; within stems, T1, T3, and T5 were significantly lower than T2, T4, and T6. Leaves showed significantly higher DPPH capacity than stems overall (organ effect, *p* < 0.0001), but the magnitude of this difference varied significantly with fertilization treatment (organ × treatment interaction, *p* = 0.0010).

DPPH radical-scavenging capacity displayed a different response pattern compared to CUPRAC (Figure 6). In leaves, DPPH activity ranged from 0.20 ± 0.02 to 0.40 ± 0.06 µmol TE g^−1^ DW, with the highest value observed in T4, corresponding to the combined application of 50% manure and 50% NPK. In stems, DPPH activity remained markedly lower, ranging from 0.01 ± 0.00 to 0.08 ± 0.03 µmol TE g^−1^ DW, with the highest values observed in T2 and T6. The divergence between CUPRAC and DPPH responses indicates that cultivation treatment influences reducing capacity and radical-scavenging activity through partially distinct chemical drivers. A two-way ANOVA on natural-log-transformed DPPH data (which was required to meet the normality assumption) confirmed organ as a highly significant determinant (*p* < 0.0001); in contrast to TFC, CUPRAC, or FRAP, the organ × treatment interaction was also significant for DPPH (*p* = 0.0010), indicating that the magnitude of the leaf-over-stem advantage varied by fertilization treatment. Simple-effects tests showed that leaves significantly exceeded stems in five out of the six treatments (T1–T5) but not in T6.

**Figure 7:**
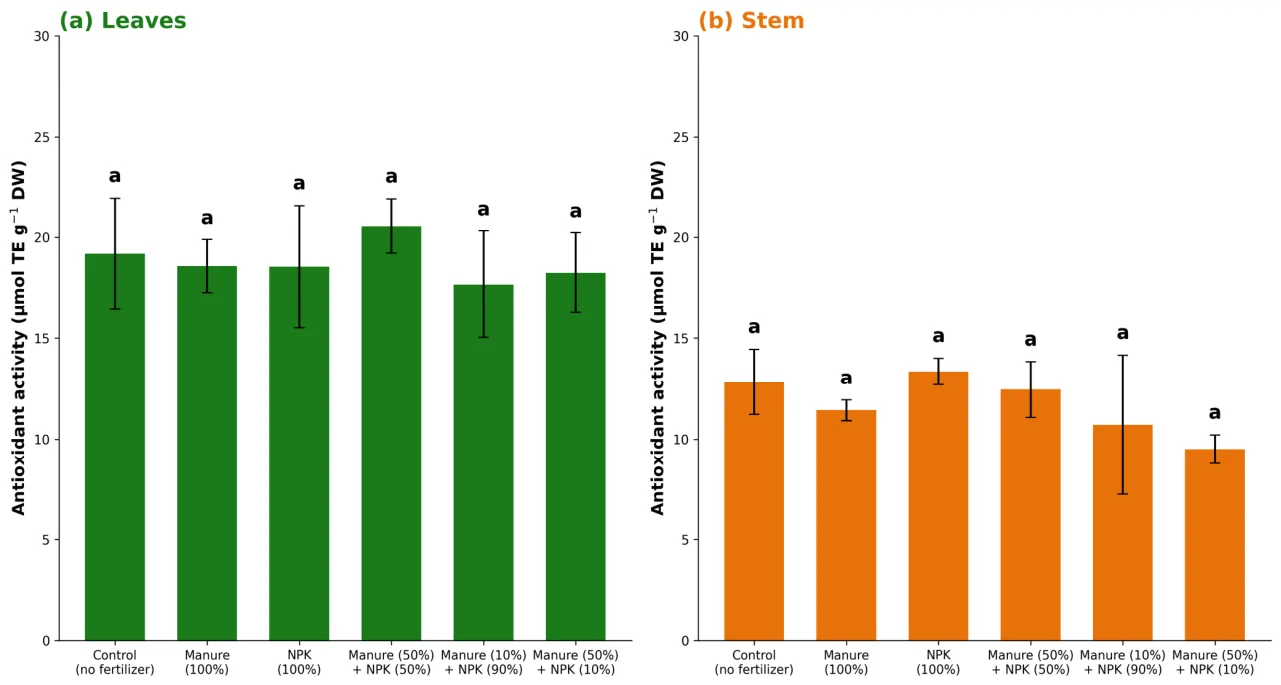
Ferric reducing antioxidant power (FRAP) of cultivated *J. gendarussa* leaf and stem extracts obtained using the corrected optimized extraction condition. Treatment codes are as described in Figure 4. Values are mean ± SEM (*n* = 3 independent extractions). Bars sharing the same letter within a panel are not significantly different (Tukey’s HSD, α = 0.05, pooled two-way ANOVA error term); all bars in both panels share the letter ‘a,’ indicating no significant treatment effect was detected (*p* = 0.728). Leaves showed significantly higher FRAP than stems overall (organ effect, *p* < 0.0001), consistently across all treatments (organ × treatment interaction not significant, *p* = 0.961).

FRAP values of the cultivated leaf and stem extracts were also determined (Figure 7). Leaf FRAP values ranged from 17.67 ± 2.63 to 20.57 ± 2.34 µmol TE g^−1^ DW, while stem values ranged from 9.51 ± 1.23 to 13.35 ± 1.14 µmol TE g^−1^ DW. A two-way ANOVA confirmed organ as a highly significant determinant of FRAP (*p* < 0.0001), with neither the treatment effect (*p* = 0.728) nor the organ × treatment interaction (*p* = 0.961) reaching significance, indicating a consistent leaf-over-stem FRAP advantage across all six fertilization treatments.

**Figure 8:**
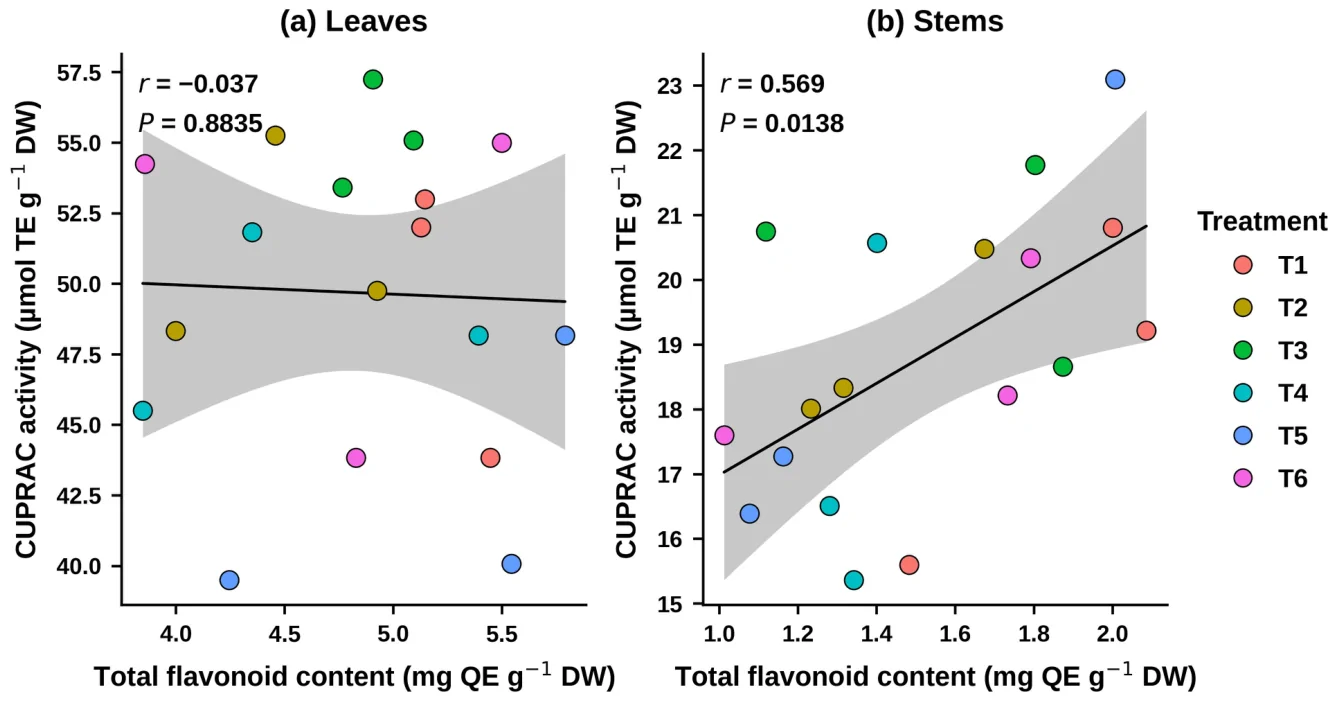
Correlation between total flavonoid content and cupric reducing antioxidant capacity (CUPRAC) antioxidant activity in cultivated *J. gendarussa* extracts: (**a**) leaves and (**b**) stems. Each point represents one biological replicate, colored by fertilization treatment (T1–T6, as defined in Figure 4). The solid line shows the linear regression fit with 95% confidence band (shaded). Pearson correlation coefficients (r) and *p*-values are shown for each organ.

**Figure 9:**
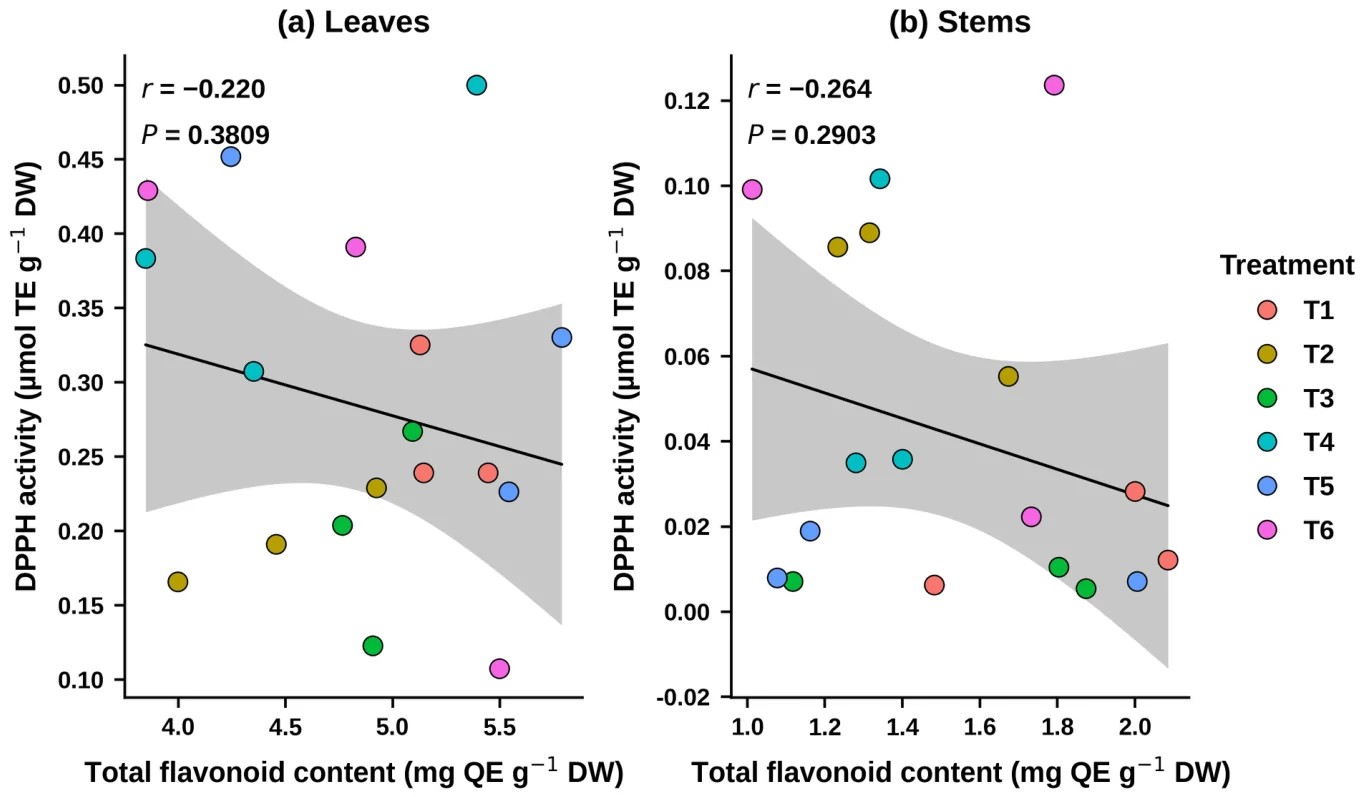
Correlation between total flavonoid content and 2,2-diphenyl-1-picrylhydrazyl (DPPH) radical-scavenging activity in cultivated *J. gendarussa* extracts: (**a**) leaves and (**b**) stems. Each point represents one biological replicate, colored by fertilization treatment (T1–T6, as defined in Figure 4). The solid line shows the linear regression fit with 95% confidence band (shaded). Pearson correlation coefficients (r) and *p*-values are shown for each organ.

**Figure 10:**
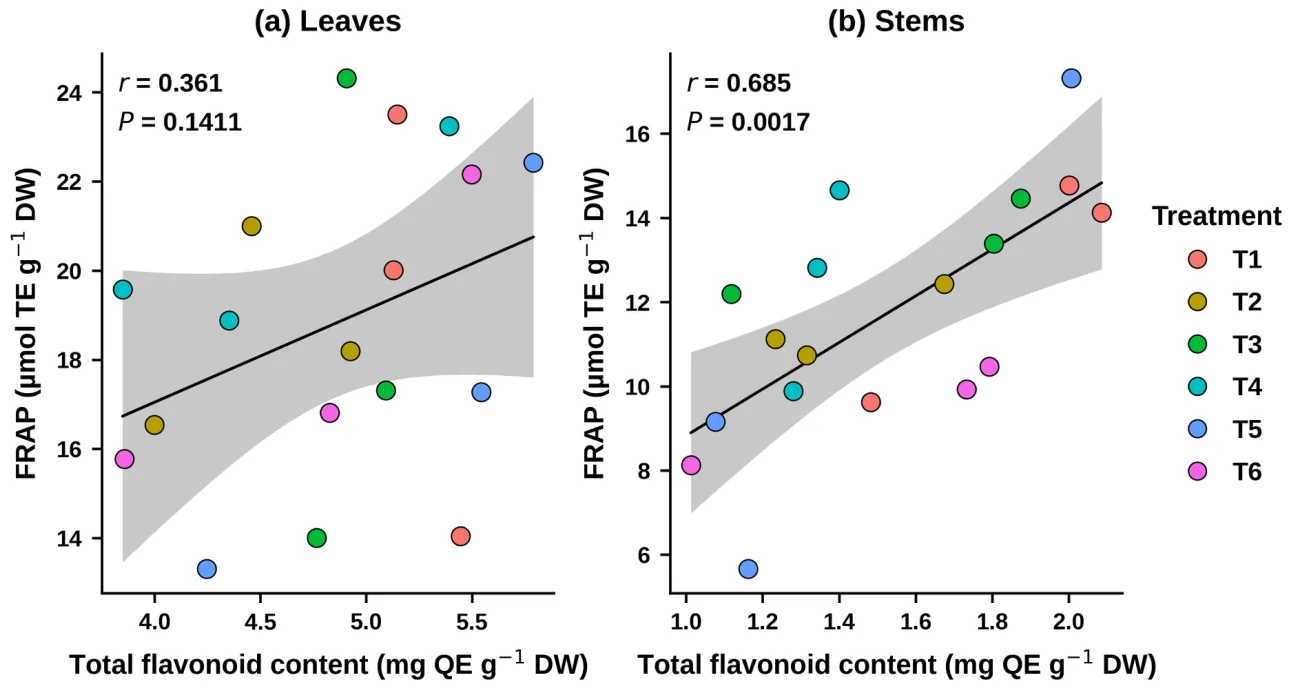
Correlation between total flavonoid content and ferric reducing antioxidant power (FRAP) in cultivated *J. gendarussa* (**a**) leaf and (**b**) stem extracts. Each point represents one biological replicate, colored by fertilization treatment (T1–T6, as defined in Figure 4). The solid line shows the linear regression fit with 95% confidence band (shaded). Pearson correlation coefficients (r) and *p*-values are shown for each organ.

Correlation analysis further clarified the relationship between flavonoid content and antioxidant activity in cultivated samples. TFC showed no significant association with CUPRAC activity in leaves (*r* = −0.037, *p* = 0.8835), whereas a moderate positive and significant correlation was observed in stems (*r* = 0.569, *p* = 0.0138) (Figure 8). In contrast, TFC showed weak negative and non-significant correlations with DPPH activity in both leaves (*r* = −0.220, *p* = 0.3809) and stems (*r* = −0.264, *p* = 0.2903) (Figure 9). TFC also showed a weak, non-significant positive correlation with FRAP in leaves (*r* = 0.361, *p* = 0.1413), but a moderate, significant positive correlation in stems (*r* = 0.685, *p* = 0.0017) (Figure 10). These results indicate that flavonoids contribute more clearly to reducing antioxidant capacity in stems than in leaves—supported by the significant TFC–CUPRAC and TFC–FRAP correlations in stems—while DPPH radical scavenging appears to be influenced by non-flavonoid constituents or by specific flavonoid subclasses not fully represented by total flavonoid quantification.

## 3. Discussion

The present study shows that extraction conditions substantially influence the recovery of flavonoids and antioxidant-associated compounds from *J. gendarussa*. The wide response ranges observed across the FFD matrix (Table 1) indicate that extraction cannot be treated as a neutral preparative step, but should instead be considered a process variable that shapes the chemical and functional profile of the resulting extract. Previous studies on *J. gendarussa* have similarly shown that solvent composition affects total phenolic content, TFC, antioxidant activity, and metabolite profiles [12], while response-surface optimization has demonstrated that ethanol concentration is an important determinant of phenolic-antioxidant recovery [13]. In this study, however, the FFD approach further clarified that the most influential effects were not limited to single variables. The liquid-to-solid ratio was the most influential factor across all three responses, either alone or in interaction with other variables: the liquid-to-solid ratio × temperature interaction (A × B) was the dominant term for TFC, while the liquid-to-solid ratio alone (A) was overwhelmingly dominant for both DPPH radical-scavenging capacity and FRAP (Table 2), indicating that the volume of solvent relative to sample mass was the primary determinant of extraction efficiency for all measured responses.

The dominance of the liquid-to-solid ratio is consistent with a mass-transfer-limited extraction process, in which a larger solvent volume relative to sample mass maintains a steeper concentration gradient between the plant matrix and the surrounding solvent, promoting more complete diffusion of phenolic and flavonoid constituents outside of the plant tissue. Previous studies have reported several phenolic and flavonoid constituents in *J. gendarussa*, including quercetin, kaempferol, naringenin, and apigenin-related compounds [10,11].

The negative A × B interaction for TFC indicates that the benefit of increasing liquid-to-solid ratio was attenuated at higher temperature, suggesting that excessive solvent dilution combined with elevated temperature does not further improve flavonoid recovery—consistent with the corrected optimal condition favoring a comparatively mild temperature (30 °C) rather than an elevated one. The ethanol concentration × pH interaction (C × E), while no longer the dominant term for TFC, remained statistically significant for both TFC and FRAP, indicating a secondary but important role for solvent polarity and extraction-medium acidity. Notably, the drastic loss of TFC along with a simultaneous increase in FRAP values observed under the corner-point condition combining the highest temperature with the lowest pH (Run 18: 100 mL g^−1^, 80 °C, 20% ethanol, 300 min, pH 2) indicates a structural transformation pathway. Strong mineral acidity at high temperatures is known to trigger the acid hydrolysis of classic flavonoid *O*-glycosides into their corresponding aglycones or drive thermal degradation. While such degradation processes inherently reduce the total concentration of intact flavonoids detectable by the aluminium chloride colorimetric assay, the resulting cleaved carbohydrate fragments or modified phenolic degradation products can possess highly exposed, free reducing groups that significantly amplify electron-transfer mechanisms in the FRAP assay [30].

This pattern suggests that the C × E interaction reflects selective extraction of chemically distinct, acid- and heat-labile phenolic and flavonoid subclasses at this extreme corner point, rather than a uniform increase in all flavonoid constituents. This interpretation is consistent with studies showing that solvent composition can modify the simultaneous recovery of phenolic and flavonoid compounds from plant matrices [31]. Nevertheless, because TFC was quantified using the aluminium chloride colorimetric assay, the observed TFC values should be interpreted as group-level estimates rather than direct measurements of individual flavonoid molecules.

Although TFC and DPPH were both strongly governed by the liquid-to-solid ratio, the distinct term structures underlying each response indicate that antioxidant capacity in *J. gendarussa* cannot be represented by a single assay. The DPPH assay is widely used to estimate radical-scavenging capacity, but its response is affected by the reaction medium, solvent system, and the balance between hydrogen atom transfer and single electron transfer reactions [19,20]. By contrast, FRAP primarily reflects ferric-to-ferrous reducing capacity and is therefore more closely associated with electron-transfer potential [20]. This difference may explain why extraction conditions that favored DPPH activity did not necessarily produce the highest FRAP response. Comparative studies also emphasize that radical-scavenging and reducing-power assays can rank plant extracts differently because each assay responds to different antioxidant mechanisms and compound classes [21,22,23]. Therefore, DPPH and FRAP should be interpreted as complementary endpoints rather than interchangeable measures of antioxidant activity. In the present study, DPPH and FRAP were both overwhelmingly dominated by the liquid-to-solid ratio alone rather than by temperature-related interactions, indicating that mass-transfer-driven recovery of both radical-scavenging and reducing compounds was primarily governed by solvent volume relative to sample mass rather than by thermal energy input.

Although temperature was not the dominant factor for FRAP, it retained a secondary, statistically significant role through its interaction with extraction time (B × D). Elevated temperature can improve extraction efficiency by increasing molecular diffusion, reducing solvent viscosity, and facilitating the release of soluble antioxidant compounds from plant tissues [7,9]. However, the effect of temperature is not necessarily linear, because excessive heating or prolonged exposure may also promote oxidation, degradation, or transformation of thermolabile phenolic constituents [32]. In the present study, the highest FRAP value was obtained under high temperature along with prolonged extraction at acidic pH, whereas the same condition produced the lowest TFC. This pattern suggests that FRAP may have been influenced by non-flavonoid reducing compounds or by compounds that remained extractable under harsher conditions. However, because metabolite profiling was not performed for each FFD run, this explanation should be interpreted cautiously. Overall, the data indicate that the liquid-to-solid ratio was the dominant factor governing recovery of both flavonoid and ferric-reducing constituents, with temperature, ethanol concentration, and pH contributing secondary, response-specific effects through their interactions with liquid-to-solid ratio.

The contrasting response patterns among TFC, DPPH, and FRAP highlight the limitation of optimizing *J. gendarussa* extraction using a single analytical endpoint. Conditions that maximize one response may not necessarily maximize another, particularly when the measured responses reflect different chemical properties. Therefore, the desirability-based multi-response approach was useful for identifying a balanced extraction condition rather than an isolated maximum for TFC, DPPH, or FRAP alone. Similar optimization studies have shown that statistically designed extraction models can support the selection of practical operating conditions when multiple phytochemical and antioxidant responses must be considered simultaneously [13,33,34,35]. In the present study, the optimized condition was experimentally validated, and the observed values remained within the prediction intervals (Table 4), indicating that the model was suitable for predicting the measured extraction responses. However, this optimum should be interpreted as a response-specific optimum for the selected assays and extraction domain, not as a universal optimum for all metabolites or biological activities. The validated protocol nevertheless provides a useful basis for producing more reproducible *J. gendarussa* extracts and for subsequent chemical profiling and cultivation-based comparison.

The UHPLC-HRMS-based profile of the optimized extract provided chemical support for the response patterns observed during extraction optimization. The detection of 52 curated peaks, including organic acids, sugar-related metabolites, amino-acid derivatives, a flavonoid *C*-glycoside, phenolic acid, hydroxy fatty acids, and oxygenated fatty acids or oxylipins, indicates that the optimized condition recovered a chemically diverse metabolite pool (Figure 3; Table 5). The putative annotation of an apigenin di-*C*-pentoside-type *C*-glycosylflavone (candidate gendarusin A/B; specific regiochemistry not confirmed by MS/MS alone) is consistent with a previous report that *J. gendarussa* contains apigenin-related flavonoid glycosides [10]. At the same time, the presence of mevalonic acid, azelaic acid, organic acids, and oxylipin-related metabolites suggests that non-flavonoid constituents may also contribute to the antioxidant responses measured in this study. This interpretation is consistent with the divergence between TFC, DPPH, and FRAP, because TFC alone cannot fully represent the chemical complexity of the optimized extract. However, these UHPLC-HRMS results should be interpreted cautiously. The metabolites were putatively annotated based on HRMS data, and relative peak areas do not represent absolute concentrations because ionization efficiency differs among compound classes. Following metabolomics annotation-confidence principles, definitive identification and quantification would require confirmation using authentic standards [24,25].

Interestingly, the untargeted metabolomic profile also indicated that a putative apigenin-type *C*-glycosylflavone (candidate gendarusin A/B, formula C_25_H_26_O_13_) accounted for a substantial proportion of the profiled features under the corrected optimal extraction condition (30 °C, pH 2). Diagnostic MS/MS fragments (sequential neutral losses of water and a cross-ring glycosidic fragment) support a *C*-glycosylflavone assignment, though the specific regiochemistry could not be established by MS/MS alone and would require an authentic standard or NMR data for confirmation. As a class, aryl-C-glycosides feature a direct carbon–carbon (Csp3–Csp2) covalent bond between the anomeric carbon of the sugar and the aromatic ring of the flavonoid skeleton, a linkage markedly more resistant to acid hydrolysis than the hemiacetal linkage of *O*-glycosides; this may help to explain its recovery even under the strongly acidic (pH 2) condition of the corrected optimum, independent of any thermal-stability argument since the corrected optimum uses a comparatively mild 30 °C rather than an elevated temperature [36].

Application of the optimized extraction protocol to cultivated *J. gendarussa* further showed that antioxidant-related traits were influenced by plant organ and fertilization regime. Leaves consistently contained higher TFC and antioxidant activity than stems, indicating that leaf tissue was the main site of flavonoid accumulation under the tested cultivation conditions (Figure 4, Figure 5, Figure 6 and Figure 7). Notably, the highest numerical TFC was observed in unfertilized leaves, while T5 resulted in a comparable value, suggesting that additional fertilizer input did not significantly increase total flavonoid accumulation in leaf tissue. This pattern may reflect a shift in plant metabolic allocation: under limited nutrient input, plants may allocate relatively more carbon toward secondary metabolites, such as flavonoids, whereas higher nutrient availability may favor growth-related primary metabolism rather than proportional increases in phenolic accumulation [26,27,37]. However, because the differences among leaf fertilization treatments were limited, this result should be interpreted as a treatment-associated tendency rather than definitive evidence that fertilizer omission enhances flavonoid biosynthesis. In contrast, antioxidant responses varied according to assay type: the highest CUPRAC activity was found under NPK treatment, whereas the highest DPPH activity occurred in the presence of the combined 50% manure + 50% NPK condition; FRAP, by contrast, showed no significant treatment-related variation in either organ (two-way ANOVA, treatment *p* = 0.728). These results suggest that fertilization may alter antioxidant-related chemistry in an endpoint- and mechanism-specific manner, possibly by affecting different metabolite groups rather than simply increasing TFC. In *J. gendarussa*, nitrogen-related cultivation has also been reported to influence metabolite profiles and antioxidant capacity, supporting the view that agronomic inputs can modify the phytochemical quality of this species [28].

The correlation analysis further supports the view that TFC alone does not fully explain antioxidant performance in cultivated *J. gendarussa*. It was not significantly correlated with CUPRAC activity in leaves, whereas a moderate positive and significant correlation was observed in stems (Figure 8). This organ-specific relationship suggests that stem flavonoids may contribute more consistently to reducing capacity, while leaf antioxidant activity may be influenced by a broader mixture of metabolites. In contrast, TFC showed weak negative and non-significant correlations with DPPH activity in both leaves and stems (Figure 9), indicating that radical-scavenging capacity was not directly proportional to total flavonoid concentration. TFC also showed a moderate, significant positive correlation with FRAP in stems (*r* = 0.685) but not in leaves (Figure 10), mirroring the organ-specific pattern observed for CUPRAC and further supporting a stronger flavonoid contribution to reducing-capacity assays specifically in stem tissue. This result is reasonable because colorimetric TFC represents a broad group-level estimate, whereas antioxidant assays are influenced by compound structure, redox potential, hydroxylation pattern, and matrix composition [20,37]. In addition, the UHPLC-HRMS profile of the optimized extract demonstrated that the validated extraction condition could recover multiple metabolite classes beyond flavonoids, including organic acids, phenolic acid, fatty-acid derivatives, and oxylipin-like metabolites. Although this profile was not generated separately for each cultivated sample, it supports the broader interpretation that antioxidant activity in *J. gendarussa* extracts may arise from several metabolite classes rather than from TFC alone. Therefore, the antioxidant activity of cultivated *J. gendarussa* should be interpreted as the combined outcome of flavonoid and non-flavonoid constituents, depending on plant organ, cultivation treatment, and assay mechanism.

Taken together, the extraction optimization, UHPLC-HRMS profiling, and cultivation-based evaluation indicate that antioxidant functionality in *J. gendarussa* is shaped by both extraction chemistry and plant-production conditions. The FFD approach identified an extraction condition that balanced TFC, DPPH, and FRAP responses, while the metabolite profile showed that the optimized extract contained chemically diverse constituents, including flavonoid and non-flavonoid metabolites. Application of the optimized protocol to cultivated samples further showed that leaves were generally richer in flavonoids and antioxidant activity than stems, but fertilization effects depended on the analytical endpoint. These findings support the use of standardized extraction as a necessary step for comparing *J. gendarussa* materials across experimental or production systems. However, several limitations should be noted. The UHPLC-HRMS annotations were putative, relative peak areas were not equivalent to absolute concentrations, and metabolite profiling was not performed separately for each cultivated treatment. Future studies should therefore combine the optimized extraction protocol with targeted quantification of key metabolites, treatment-specific metabolomics, and cell-based antioxidant assays to determine whether the observed in vitro antioxidant profiles are reflected in biological systems.

Lastly, a numerical difference in the DPPH assay response between the optimization and cultivation phases is worth noting. Confirmatory extraction under the corrected optimal condition yielded a DPPH radical-scavenging activity of 12.30 ± 0.02 µmol TE g^−1^ DW. Values observed during the cultivation experiments with the leaf material were between 0.20 and 0.40 µmol TE g^−1^ DW—substantially closer than under the originally reported (uncorrected) optimum, though a difference remains. This residual difference was largely due to the different raw botanical matrices that had different origins, developmental ages, and microenvironment histories. The optimization phase used an established, highly concentrated uniform repository pool of older, field-grown materials, whereas the cultivation experiment used young plant tissue of cuttings grown in short, controlled, and temporary polybag systems. It has been well established that changes in age, growth medium, and root-zone restriction alter steady-state specialized secondary metabolite accumulation profiles [38,39,40]. These changes also lead to a shift in the radical-scavenging absolute scale, and do not negate the relative phenotypic trends observed between fertilization treatments.

## 4. Materials and Methods

### 4.1. Plant Material and Sample Preparation for Extraction Optimization

**Figure 11:**
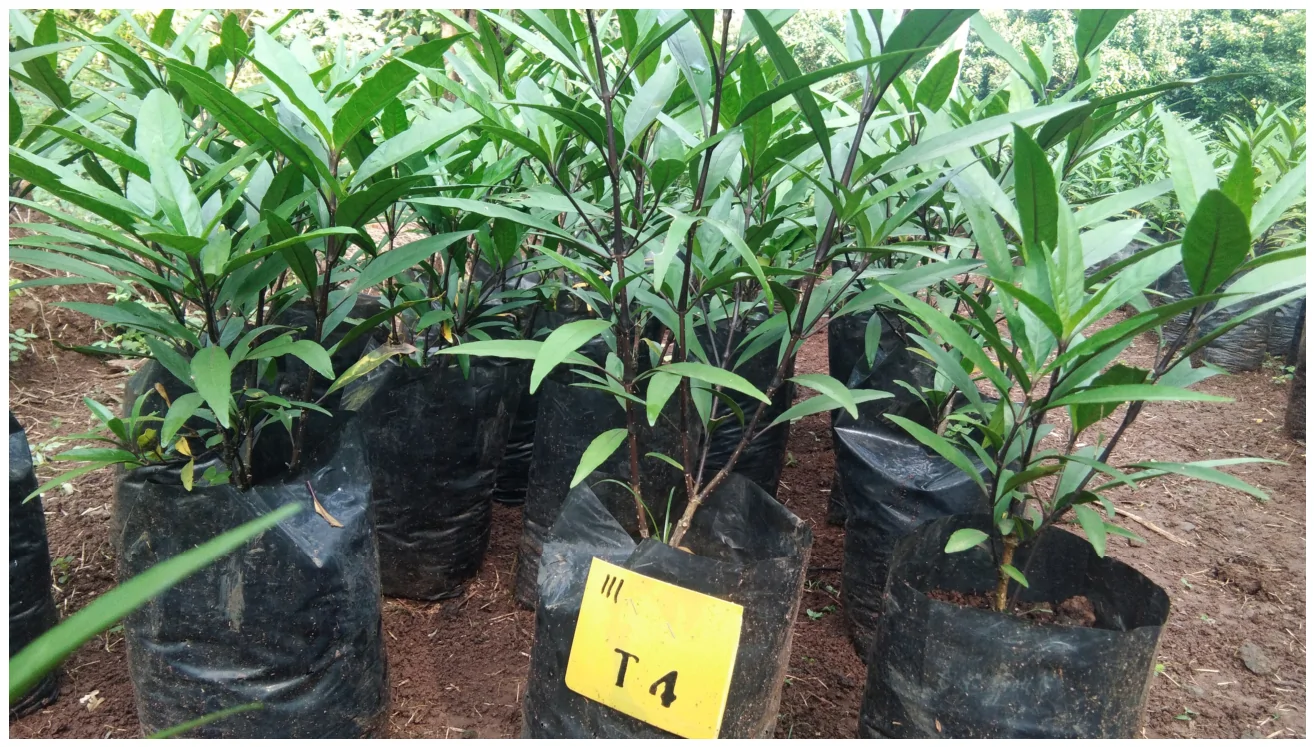
*Justicia gendarussa* plants (fertilization treatment T4) cultivated in polybags at the nursery of the Tropical Biopharmaca Research Center, Bogor, West Java, Indonesia.

Leaves of *J. gendarussa* were obtained from the Tropical Biopharmaca Research Center, Bogor, West Java, Indonesia. Fresh leaves were sorted to remove non-target materials, washed under running water, drained, and oven-dried at 45 °C (Memmert UN30, Schwabach, Germany). Residual impurities were removed from the dried leaves, which were subsequently ground using an Ossel FFC-15 disc mill (3400 rpm, 1.5 kW; Ossel, China) and passed through an 80-mesh sieve to obtain a uniformly sieved powder. The resulting *J. gendarussa* leaf powder was used for all extraction optimization experiments (Figure 11).

### 4.2. Experimental Design and Extraction Procedure

#### 4.2.1. Half-Fractional Factorial Design

A two-level half-FFD was generated using Stat-Ease 360 software (version 23.1.4.0; Stat-Ease Inc., Minneapolis, MN, USA) to evaluate the extraction process. Five independent variables were investigated: liquid-to-solid ratio (A), extraction temperature (B), ethanol concentration (C), extraction time (D), and extraction pH (E). The experimental domain and actual factor levels are shown in Table 6. The final design comprised 16 factorial runs and three independently prepared center-point runs, yielding a total of 19 randomized experimental runs. Each factorial condition was represented by a single independently performed extraction, whereas the center-point condition was independently repeated three times. The center-point runs were used to estimate pure error and assess model curvature. Each extract was analyzed using three technical analytical measurements, and the mean was used as the run-level response for statistical modeling. The full experimental matrix and observed responses are presented in Table 1.

#### 4.2.2. Extraction Procedure for Optimization

Extractions were performed using a temperature-controlled water bath (Memmert, Schwabach, Germany). For each experimental run, 1.0 g of dried *J. gendarussa* leaf powder was mixed with 10, 55, or 100 mL of aqueous ethanol, corresponding to the liquid-to-solid ratio specified in the experimental design. The ethanol concentration, extraction temperature, extraction time, and pH were adjusted according to the corresponding experimental condition. Before extraction, the pH of the extraction solvent was adjusted to the designated value using hydrochloric acid (HCl) or sodium hydroxide (NaOH), as required. The pH meter (AD1000, Adwa Instruments, Szeged, Hungary) was calibrated with standard pH 4.01 and 7.00 buffer solutions before each measurement session, and the pH of the extraction solvent was adjusted using 1 mol L^−1^ HCl or 1 mol L^−1^ NaOH, added dropwise until the designated pH value was reached. After extraction, the mixtures were filtered, and the filtrates were immediately collected for the determination of TFC and antioxidant capacity. All chemicals and reagents used in this study were of analytical grade and were purchased from Sigma-Aldrich (St. Louis, MO, USA).

### 4.3. Determination of Total Flavonoid Content and Antioxidant Capacity

#### 4.3.1. Total Flavonoid Content

TFC was quantified using a modified aluminium chloride colorimetric assay [1]. In a 96-well microplate, 10 µL of extract was mixed with 120 µL of distilled water, 10 µL of 10% (*w*/*v*) AlCl_3_, 10 µL of glacial acetic acid, and 50 µL of ethanol. The mixture was incubated for 30 min in the dark at room temperature. Absorbance was measured at 415 nm using a microplate spectrophotometer (SPECTROstarNano, BMG LABTECH, Offenburg, Germany). Quercetin standards ranging from 25 to 500 ppm were used to construct the calibration curve, and a 0-ppm reagent blank was used for blank correction. The calibration equation was *y* = 0.0014*x* + 0.0157, with *R*^2^ = 0.9852, where *y* represents the blank-corrected absorbance and *x* represents the quercetin concentration. Sample absorbance values were blank-corrected, and dilution factors of 1 or 5 were applied as appropriate and incorporated into the calculations. Results were expressed as milligrams of quercetin equivalents per gram of dry weight (mg QE g^−1^ DW). Each extract was analyzed in three technical analytical replicates, and the mean value was used as the run-level response for statistical modeling.

#### 4.3.2. DPPH Radical-Scavenging Capacity

DPPH radical-scavenging capacity was determined according to a modified method adapted from Nurcholis et al. [41]. Briefly, 100 µL of extract was mixed with 100 µL of DPPH ethanolic solution in a clear 96-well microplate. The mixture was incubated for 30 min in the dark at room temperature, and absorbance was measured at 515 nm. Antioxidant capacity was calculated using a Trolox standard curve and expressed as micromoles of Trolox equivalents per gram of dry weight (µmol TE g^−1^ DW). All measurements were conducted in triplicate.

#### 4.3.3. Ferric Reducing Antioxidant Power

FRAP was evaluated according to the method described by Makkiyah et al. [42], with minor modification. The FRAP working reagent was freshly prepared by mixing acetate buffer, FeCl_3_·6H_2_O solution, and 2,4,6-tripyridyl-*s*-triazine solution at a 10:1:1 (*v*/*v*/*v*) ratio and warmed to 37 °C before use. Aliquots of extract or Trolox standard solution were added to FRAP reagent in a microplate. After incubation at 37 °C in the dark, absorbance was measured at 593 nm. Results were expressed as µmol TE g^−1^ DW. Measurements were performed in triplicate.

### 4.4. Statistical Modeling, Multi-Response Optimization, and Experimental Validation

Statistical modeling and multi-response optimization were performed using Stat-Ease 360 software (v. 23.1.4.0; Stat-Ease Inc., Minneapolis, MN, USA). For each response variable, models incorporating main effects and two-factor interactions were constructed. Model adequacy and the statistical significance of individual model terms were evaluated using analysis of variance (ANOVA), with statistical significance set at *p* < 0.05.

The optimum extraction condition was selected using a desirability-based multi-response approach aimed at simultaneously maximizing TFC, DPPH radical-scavenging capacity, and FRAP. The predicted optimum was experimentally validated by conducting independent extractions under the selected condition. The confirmatory extract was analyzed in triplicate (three technical replicates), and model predictability was evaluated using relative prediction error:

$$\mathrm{Prediction}\;\mathrm{error}\;(\%)=\frac{\left[\mathrm{Experimental mean}-\mathrm{Predicted value}\right]}{\mathrm{Predicted value}}\times 100$$

### 4.5. UHPLC-Q-Orbitrap HRMS Analysis of the Optimized Extract

The extract prepared under the corrected optimal condition was subjected to UHPLC-HRMS/MS analysis for metabolite profiling, using a Vanquish UHPLC system coupled to a Q Exactive Plus Orbitrap high-resolution mass spectrometer (Thermo Fisher Scientific, Waltham, MA, USA). Prior to injection, the extract was filtered through a 0.2 µm PTFE membrane filter and diluted with methanol to an appropriate final volume. Chromatographic separation was performed using an Accucore C18 column (100 × 2.1 mm, 1.5 µm particle size; Thermo Fisher Scientific, Waltham, MA, USA). The mobile phase consisted of 0.1% formic acid in water as solvent A and 0.1% formic acid in acetonitrile as solvent B. Gradient elution was applied as follows: 0–1 min, 5% B; 1–25 min, 5–95% B; 25–28 min, 95% B; and 28–33 min, 95–5% B. The flow rate was 0.2 mL min^−1^, the injection volume was 2 µL, and the column temperature was maintained at 30 °C.

Mass spectrometric detection was performed using heated electrospray ionization (HESI) in positive ion mode, with full-scan MS acquired at 70,000 resolution over an *m/z* range of 100–1500, and data-dependent MS/MS (dd-MS^2^, Top 5) acquired using stepped higher-energy collisional dissociation (HCD) at normalized collision energies of 18, 35, and 53. Raw UHPLC-HRMS/MS data were processed using Compound Discoverer 3.2 software (Thermo Fisher Scientific, Waltham, MA, USA) for peak detection, retention-time alignment, feature extraction, mzCloud/ChemSpider spectral and formula matching (mass tolerance 5 ppm), and putative metabolite annotation, followed by manual verification of MS/MS spectra for compounds with ambiguous or conflicting annotations. Curated metabolite features were exported for tabulation and visualization. Metabolites were reported as putatively annotated compounds at Metabolomics Standards Initiative (MSI) confidence Level 2 (mzCloud spectral match) or Level 3 (accurate mass and formula match only); definitive structural confirmation was not assigned because authentic standards were not used.

### 4.6. Cultivation Experiment and Application of the Optimized Extraction Protocol

To evaluate the applicability of the optimized extraction condition to cultivated plant material, leaf and stem samples of cultivated *J. gendarussa* were obtained from plants grown at the Tropical Biopharmaca Research Center, IPB University, Bogor, Indonesia. The experimental site is located at approximately 6°3′49″ S and 106°42′57″ E, at an elevation of 141 m above sea level.

Plants were propagated from 15 cm stem cuttings and initially grown in 10 × 15 cm polybags for two months. Seedlings were subsequently transferred to 25 × 30 cm polybags for fertilization treatments. The cultivation experiment followed a randomized complete block design with six fertilization regimes: T1, control without fertilizer; T2, 100% manure; T3, 100% NPK; T4, 50% manure + 50% NPK; T5, 10% manure + 50% NPK; and T6, 50% manure + 10% NPK. Each treatment was replicated three times, and each replication consisted of 20 plants. Plants were cultivated for four months before sample collection.

Leaves and stems were harvested separately, dried, ground, and extracted using the corrected optimized extraction conditions: liquid-to-solid ratio of 100 mL g^−1^, extraction temperature of 30 °C, ethanol concentration of 20% (*v*/*v*), extraction time of 30 min, and pH 2. The resulting extracts were filtered and used for TFC, CUPRAC, DPPH, and FRAP analyses.

### 4.7. CUPRAC Antioxidant Capacity of Cultivated Samples

CUPRAC was measured using a modified microplate method based on the Cu(II)-neocuproine reducing system [43]. Briefly, aliquots of leaf or stem extract were mixed with CuCl_2_ solution, neocuproine solution, and ammonium acetate buffer in a 96-well microplate. The mixture was incubated for 30 min in the dark at room temperature, and absorbance was measured at 450 nm using a SPECTROstarNano microplate spectrophotometer. Trolox was used as the reference standard, and results were expressed as µmol TE g^−1^ DW.

### 4.8. Statistical Analysis and Figure Preparation

All analytical measurements were performed in triplicate. Data from extraction optimization and validation were reported as mean ± standard deviation (SD). Data from cultivated samples were reported as mean ± standard error of the mean (SEM). For the cultivation experiment, a two-way ANOVA (organ × fertilization treatment) followed by Tukey’s HSD post hoc test was used to compare responses across plant organs and fertilization treatments. Differences were considered statistically significant at *p* < 0.05.

Correlation analyses between TFC and antioxidant capacity were performed separately for leaves and stems using Pearson correlation analysis. Statistical summaries, ANOVA, Tukey’s tests, and correlation analyses for the cultivation experiment were generated using R (version 4.5.2; R Foundation for Statistical Computing, Vienna, Austria) in RStudio (version 2025.09.0+387; Posit Software, PBC, Boston, MA, USA). The cultivation-related figures were prepared using the ggplot2 package and exported as high-resolution TIFF files at 600 dpi using the ragg package.

For metabolite-profile visualization, curated UHPLC-HRMS features exported from Compound Discoverer were plotted using their retention time and relative peak area values. A reconstructed UHPLC-HRMS chromatogram was generated in RStudio using ggplot2, with peak numbers corresponding to the putatively annotated metabolites listed in Table 5 and colors indicating metabolite classes. The chromatogram was exported as a high-resolution TIFF file at 600 dpi using the ragg package. Additional graphical visualizations for the extraction optimization study were generated using GraphPad Prism (v. 11.0.0, GraphPad Software, San Diego, CA, USA).

## 5. Conclusions

This study showed that extraction conditions influenced the measured flavonoid content and antioxidant capacity of *J. gendarussa* leaf extracts. The half-FFD indicated that the liquid-to-solid ratio × temperature interaction was the dominant determinant of TFC, whereas the liquid-to-solid ratio alone dominated both DPPH radical-scavenging capacity and FRAP. These results suggest that the measured antioxidant responses were affected by different extraction-related factors.

A desirability-based approach identified an operational extraction condition of 100 mL g^−1^ liquid-to-solid ratio, 30 °C extraction temperature, 20% ethanol, 30 min extraction time, and pH 2. Confirmatory extraction showed that the predicted and observed values were in excellent agreement (0.10–1.54% error) for the responses evaluated in this study. UHPLC-Q Exactive Plus Orbitrap HRMS profiling of the optimized extract detected 52 putatively annotated metabolites, including a candidate gendarusin A/B (apigenin di-C-arabinoside)-type C-glycosylflavone, amino acids, organic acids, and oxygenated fatty acid derivatives. Because these annotations were not confirmed using authentic standards, they should be interpreted as preliminary metabolite assignments.

When the optimized protocol was applied to cultivated *J. gendarussa* samples, leaves showed significantly higher TFC, CUPRAC, and FRAP than stems regardless of fertilization treatment (two-way ANOVA, organ effect *p* < 0.0001 for each assay; organ × treatment interaction not significant). For DPPH, leaves also showed significantly higher capacity than stems overall (*p* < 0.0001), but the magnitude of this difference varied significantly with fertilization treatment (organ × treatment interaction, *p* = 0.0010). Overall, the results provide a validated extraction condition for the measured responses and indicate that further targeted metabolite quantification and biological assays are needed to support broader standardization of *J. gendarussa* extracts.

## Figures and Tables

**Table 1:** Experimental matrix and observed responses for the extraction of *Justicia gendarussa* leaves.

Run	A	B	C	D	E	TFC (mg QE g^−1^ DW)	DPPH (µmol TE g^−1^ DW)	FRAP (µmol TE g^−1^ DW)
1	10	80	20	30	2	4.54 ± 0.039	0.20 ± 0.007	31.50 ± 1.202
2	10	30	80	30	2	3.75 ± 0.042	0.13 ± 0.004	25.55 ± 0.692
3	10	30	20	300	2	5.08 ± 0.053	0.43 ± 0.014	36.24 ± 1.996
4	55	55	50	165	4	6.91 ± 0.194	1.42 ± 0.002	45.94 ± 2.239
5	100	30	80	30	6	7.62 ± 0.149	26.64 ± 0.270	35.42 ± 0.235
6	55	55	50	165	4	7.06 ± 0.216	1.49 ± 0.001	53.98 ± 0.366
7	10	30	20	30	6	4.21 ± 0.022	0.47 ± 0.056	19.45 ± 0.160
8	100	80	20	30	6	10.09 ± 0.258	2.55 ± 0.037	37.70 ± 0.231
9	100	80	80	30	2	8.40 ± 0.149	12.56 ± 0.056	65.18 ± 1.175
10	10	80	20	300	6	12.05 ± 0.094	0.30 ± 0.001	55.01 ± 0.308
11	10	30	80	300	6	3.54 ± 0.049	0.17 ± 0.002	30.34 ± 0.627
12	10	80	80	300	2	10.81 ± 0.149	0.28 ± 0.002	52.60 ± 0.089
13	100	80	80	300	6	12.28 ± 0.289	1.93 ± 0.022	133.00 ± 2.912
14	100	30	80	300	2	13.16 ± 0.446	2.95 ± 0.003	104.02 ± 0.968
15	100	30	20	30	2	17.35 ± 0.367	13.59 ± 0.507	214.20 ± 14.541
16	10	80	80	30	6	8.90 ± 0.129	0.29 ± 0.003	40.19 ± 0.160
17	100	30	20	300	6	25.57 ± 0.165	2.60 ± 0.018	146.20 ± 1.538
18	100	80	20	300	2	3.02 ± 0.247	2.19 ± 0.009	242.41 ± 0.769
19	55	55	50	165	4	7.79 ± 0.079	1.58 ± 0.011	132.21 ± 0.646

Note: Data are presented as mean ± SD. A, liquid-to-solid ratio (mL g^−1^); B, temperature (°C); C, ethanol concentration (% *v*/*v*); D, extraction time (min); E, pH; TFC, total flavonoid content; DPPH, 2,2-diphenyl-1-picrylhydrazyl radical-scavenging capacity; FRAP, ferric reducing antioxidant power; QE, quercetin equivalents; TE, Trolox equivalents; and DW, dry weight.

**Table 2:** Dominant significant model terms affecting total flavonoid content and antioxidant capacity in *Justicia gendarussa* leaf extracts.

Response	Model Term	*p*-Value	Contribution (%)
TFC	A × B (−)	0.0005	28.03
TFC	A	0.0010	22.66
TFC	B × C	0.0044	13.80
DPPH	A	<0.0001	79.65
DPPH	A × D (−)	0.0125	5.83
DPPH	D (−)	0.0298	4.16
FRAP	A	<0.0001	39.82
FRAP	D	0.0061	9.22
FRAP	A × C (−)	0.0085	8.07

Note: A, liquid-to-solid ratio; B, extraction temperature; C, ethanol concentration; D, extraction time; TFC, total flavonoid content; DPPH, 2,2-diphenyl-1-picrylhydrazyl radical-scavenging capacity; and FRAP, ferric reducing antioxidant power. Only statistically significant model terms are presented.

**Table 3:** Top-ranked multi-response optimization solutions for *Justicia gendarussa* leaf extraction.

Rank	A	B	C	D	E	TFC (mg QE g^−1^ DW)	DPPH (µmol TE g^−1^ DW)	FRAP (µmol TE g^−1^ DW)	Desirability
1	100.00	30.00	20.00	30.00	2.00	16.451	12.112	191.279	0.719
2	99.97	30.00	20.00	32.27	2.11	16.509	11.948	188.812	0.717
3	100.00	30.00	20.26	51.03	2.02	16.417	10.803	192.734	0.713
4	100.00	30.00	20.00	45.46	2.26	16.605	11.135	186.499	0.711
5	100.00	30.00	20.95	30.00	2.40	16.635	12.112	179.675	0.708

Note: A, liquid-to-solid ratio (mL g^−1^); B, extraction temperature (°C); C, ethanol concentration (% *v/v*); D, extraction time (min); E, extraction pH; TFC, total flavonoid content; DPPH, 2,2-diphenyl-1-picrylhydrazyl radical-scavenging capacity; FRAP, ferric reducing antioxidant power; QE, quercetin equivalents; TE, Trolox equivalents; and DW, dry weight. Solutions were ranked according to overall desirability for simultaneous response maximization.

**Table 4:** Experimental validation of the optimized extraction condition for *Justicia gendarussa* leaf extracts.

Response	Predicted Value	95% Prediction Interval	Experimental Value (Mean ± SD, *n* = 3)	Prediction Error (%)
TFC (mg QE g^−1^ DW)	16.451	11.467–21.435	16.434 ± 0.303	0.10
DPPH (µmol TE g^−1^ DW)	12.112	4.207–25.601	12.299 ± 0.016	1.54
FRAP (µmol TE g^−1^ DW)	191.279	140.510–242.049	190.558 ± 2.577	0.38

Note: Validation was performed at A = 100 mL g^−1^, B = 30 °C, C = 20% (*v*/*v*) ethanol, D = 30 min, and E = pH 2. Prediction error (%) was calculated as |experimental mean − predicted value|/predicted value × 100. TFC, total flavonoid content; DPPH, 2,2-diphenyl-1-picrylhydrazyl radical-scavenging capacity; FRAP, ferric reducing antioxidant power; QE, quercetin equivalents; TE, Trolox equivalents; and DW, dry weight.

**Table 5:** Putatively annotated metabolites detected in the optimized *Justicia gendarussa* extract by Ultra-high-performance liquid chromatography coupled to quadrupole-Orbitrap high-resolution mass spectrometry.

Peak No.	Putatively Annotated Metabolite	Putative Class	Formula	RT (min)	Calc. MW	Δ Mass (ppm)	Relative Signal Area (%)
1	O-ureido-D-serine	Amino acid derivative	C_4_H_9_N_3_O_4_	0.952	163.0594	0.42	0.81
2	6-(α-D-glucosaminyl)-1D-myo-inositol	Inositol glycoside	C_12_H_23_NO_10_	0.999	341.1322	−0.11	0.59
3	α-Aminoadipic acid	Amino acid	C_6_H_11_NO_4_	1.059	161.0688	−0.03	0.40
4	Choline	Amine (choline)	C_5_H_13_NO	1.059	103.0999	1.68	5.69
5	Hexopyranosyl-(1→4)hexopyranosyl-(1→4)hexopyranosyl-(1→4)hexopyranose	Carbohydrate (oligosaccharide)	C_24_H_42_O_21_	1.072	666.2216	−0.38	0.53
6	DL-Carnitine	Amine/carnitine	C_7_H_15_NO_3_	1.072	161.1051	−0.65	0.35
7	Maltohexaose	Carbohydrate (oligosaccharide)	C_36_H_62_O_31_	1.076	990.3270	−0.56	0.75
8	Maltoheptaose	Carbohydrate (oligosaccharide)	C_42_H_72_O_36_	1.077	1152.3799	−0.34	0.36
9	Maltopentaose	Carbohydrate (oligosaccharide)	C_30_H_52_O_26_	1.078	828.2743	−0.49	0.50
10	L-(+)-Valine	Amino acid	C_5_H_11_NO_2_	1.090	117.0789	−0.30	28.09
11	Creatine	Guanidino metabolite (common in animals; requires confirmation in plants)	C_4_H_9_N_3_O_2_	1.091	131.0694	−0.36	0.40
12	bis-β-D-fructofuranose 1,2′:2,3′-dianhydride	Carbohydrate (sugar anhydride)	C_12_H_20_O_10_	1.096	324.1053	−1.16	2.28
13	Lactide	Suspected contaminant (lactic acid/PLA degradation product)	C_6_H_8_O_4_	1.099	144.0422	−0.67	0.36
14	DL-Glutamic acid	Amino acid	C_5_H_9_NO_4_	1.102	147.0530	−0.79	0.93
15	L-Proline	Amino acid	C_5_H_9_NO_2_	1.109	115.0634	0.84	2.48
16	D-Sucrose	Carbohydrate	C_12_H_22_O_11_	1.138	342.1159	−0.75	1.00
17	Stachydrine	Alkaloid (proline betaine)	C_7_H_13_NO_2_	1.140	143.0946	−0.26	0.31
18	2′-deoxymugineic acid	Phytosiderophore (plant-specific, Poaceae family)	C_12_H_20_N_2_O_7_	1.142	304.1269	−0.57	0.38
19	γ-Guanidinobutyrate	Amino acid derivative (GABA-related)	C_5_H_11_N_3_O_2_	1.148	145.0850	−0.50	0.36
20	Guanine	Nucleobase (purine)	C_5_H_5_N_5_O	1.164	151.0494	−0.40	0.75
21	(2S)-3-Methyl-2-({[(3S,4S,5R)-2,3,4-trihydroxy-5-(hydroxymethyl)tetrahydro-2-furanyl]methyl}amino)butanoic acid (non-preferred name)	Amino acid derivative (Amadori product, e.g., fructosyl-valine)	C_11_H_21_NO_7_	1.189	279.1316	−0.58	0.66
22	(2S)-4-Methyl-2-({[(3S,4S,5R)-2,3,4-trihydroxy-5-(hydroxymethyl)tetrahydro-2-furanyl]methyl}amino)pentanoic acid (non-preferred name)	Amino acid derivative (Amadori product, e.g., fructosyl-leucine)	C_12_H_23_NO_7_	1.225	293.1469	−1.89	0.40
23	Diethylene glycol	Suspected contaminant (solvent/plasticizer)	C_4_H_10_O_3_	1.243	106.0631	1.47	2.47
24	Hypoxanthine	Nucleobase (purine)	C_5_H_4_N_4_O	1.265	136.0384	−0.87	0.69
25	Uric acid	Purine base (metabolite)	C_5_H_4_N_4_O_3_	1.367	168.0283	−0.56	0.77
26	L-(+)-Valine	Amino acid	C_5_H_11_NO_2_	1.389	117.0789	−0.30	1.76
27	Carzenide	Sulfonated aromatic compound (tentative)	C_7_H_7_NO_4_S	1.412	201.0087	−4.37	1.69
28	5-sulfooxymethylfurfural	Furan derivative (sugar degradation product)	C_6_H_6_O_6_S	1.414	205.9876	−4.58	2.39
29	Hypoxanthine	Nucleobase (purine)	C_5_H_4_N_4_O	1.440	136.0384	−0.87	0.60
30	Diethylene glycol	Suspected contaminant (solvent/plasticizer)	C_4_H_10_O_3_	1.444	106.0631	1.47	4.89
31	L-Pyroglutamic acid	Amino acid derivative	C_5_H_7_NO_3_	1.452	129.0426	−0.27	0.51
32	DL-Tyrosine	Amino acid	C_9_H_11_NO_3_	1.507	181.0737	−0.89	0.75
33	Glutarylcarnitine	Acylcarnitine	C_12_H_21_NO_6_	1.573	275.1367	−0.55	0.51
34	(2S)-4-Methyl-2-({[(3S,4S,5R)-2,3,4-trihydroxy-5-(hydroxymethyl)tetrahydro-2-furanyl]methyl}amino)pentanoic acid (non-preferred name)	Amino acid derivative (Amadori product, e.g., fructosyl-leucine)	C_12_H_23_NO_7_	1.575	293.1469	−1.89	2.72
35	L-(+)-Leucine	Amino acid	C_6_H_13_NO_2_	1.627	131.0945	−1.22	12.17
36	(2S)-3-Phenyl-2-({[(3S,4S,5R)-2,3,4-trihydroxy-5-(hydroxymethyl)tetrahydro-2-furanyl]methyl}amino)propanoic acid (non-preferred name)	Amino acid derivative (Amadori product, e.g., fructosyl-phenylalanine)	C_15_H_21_NO_7_	2.333	327.1316	−0.59	0.91
37	DL-Phenylalanine	Amino acid	C_9_H_11_NO_2_	2.413	165.0788	−1.00	6.98
38	Kynurenine	Indole derivative (tryptophan metabolite)	C_10_H_12_N_2_O_3_	2.458	208.0847	−0.50	0.54
39	DL-Phenylalanine	Amino acid	C_9_H_11_NO_2_	2.728	165.0788	−1.00	0.65
40	N-[(4-Methoxy-1-benzofuran-5-yl)carbonyl]glycine	Phenolic/benzofuran conjugate	C_12_H_11_NO_5_	4.946	249.0634	−1.47	0.61
41	Indoleacrylic acid	Indole derivative (tryptophan metabolite)	C_11_H_9_NO_2_	5.057	187.0630	−1.56	1.10
42	DL-Tryptophan	Amino acid	C_11_H_12_N_2_O_2_	5.058	204.0895	−1.80	1.69
43	Methyl 2,3-dihydro-3-hydroxy-2-oxo-1H-indole-3-acetate	Indole derivative (auxin metabolism-related)	C_11_H_11_NO_4_	5.292	221.0686	−0.71	0.34
44	(Z)-N-[(2S)-2-Amino-5-carbamimidamido-1-hydroxypentylidene]-4-hydroxy-5-phosphononorvaline	Amino acid derivative (arginine-related, phosphate-containing)	C_11_H_24_N_5_O_7_P	7.069	369.1421	2.01	0.58
45	Methoxyindoleacetic acid	Indole derivative (tryptophan metabolite)	C_11_H_11_NO_3_	7.069	205.0737	−0.78	0.43
46	(Z)-N-[(2S)-2-Amino-5-carbamimidamido-1-hydroxypentylidene]-4-hydroxy-5-phosphononorvaline	Amino acid derivative (arginine-related, phosphate-containing)	C_11_H_24_N_5_O_7_P	7.685	369.1421	2.01	0.48
47	Methoxyindoleacetic acid	Indole derivative (tryptophan metabolite)	C_11_H_11_NO_3_	7.685	205.0737	−0.78	0.32
48	Gendarusin A/B (tentative)	Flavonoid *C*-glycoside (candidate gendarusin A/B, characteristic *J. gendarussa* marker)	C_25_H_26_O_13_	8.520	534.1363	−1.87	2.52
49	Genistein	Flavonoid (isoflavone genistein OR flavone apigenin isomer—identical mass)	C_15_H_10_O_5_	12.767	270.0525	−1.06	0.31
50	Sphinganine	Sphingolipid	C_18_H_39_NO_2_	18.778	301.2975	−1.91	0.94
51	Pheophorbide A	Pigment (chlorophyll degradation product)	C_35_H_36_N_4_O_5_	26.700	592.2684	−0.25	1.00
52	Palmitamide	Fatty acid amide	C_16_H_33_NO	28.361	255.2560	−0.85	0.32

Note: RT, retention time; and MW, molecular weight. Relative area was calculated from the curated metabolite set. All metabolites are reported as putatively annotated compounds and were not confirmed using authentic standards.

**Table 6:** Independent variables and actual levels used in the half-fractional factorial design for extraction of bioactive compounds from *Justicia gendarussa* dried leaves.

Factor	Code	Low (−1)	Center (0)	High (+1)	Unit
Liquid-to-solid ratio	A	10	55	100	mL g^−1^
Temperature	B	30	55	80	°C
Ethanol concentration	C	20	50	80	% (*v*/*v*)
Extraction time	D	30	165	300	min
pH	E	2	4	6	—

## Data Availability

The original contributions presented in this study are included in the article. Further inquiries can be directed to the corresponding author.

## Abbreviations

The following abbreviations are used in this manuscript:

ANOVA	Analysis of variance
CUPRAC	Cupric reducing antioxidant capacity
DPPH	2,2-diphenyl-1-picrylhydrazyl
DW	Dry weight
FFD	Fractional factorial design
FRAP	Ferric reducing antioxidant power
HRMS	High-resolution mass spectrometry
LPDP	Indonesian Endowment Fund for Education
MS	Mass spectrometry
MW	Molecular weight
NPK	Nitrogen, phosphorus, and potassium fertilizer
QE	Quercetin equivalents
RT	Retention time
SD	Standard deviation
SEM	Standard error of the mean
TE	Trolox equivalents
TFC	Total flavonoid content
UHPLC	Ultra-high-performance liquid chromatography
UHPLC-Q-Orbitrap HRMS	Ultra-high-performance liquid chromatography coupled to quadrupole-Orbitrap high-resolution mass spectrometry
